# Stress, anxiety, depression and sleep disturbance among healthcare professional during the COVID-19 pandemic: An umbrella review of 72 meta-analyses

**DOI:** 10.1371/journal.pone.0302597

**Published:** 2024-05-09

**Authors:** Mohammed Al Maqbali, Ahmad Alsayed, Ciara Hughes, Eileen Hacker, Geoffrey L. Dickens

**Affiliations:** 1 Fatima College of Health Sciences, Al Ain, United Arab Emirates; 2 Faculty of Pharmacy, Department of Clinical Pharmacy and Therapeutics, Applied Science Private University, Amman, Jorden; 3 Institute of Nursing and Health Research School of Health Sciences, Ulster University, Belfast, United Kingdom; 4 University of Texas MD Anderson Cancer Center, Houston, Texas, United States of America; 5 Midwifery and Health Faculty of Health and Life Sciences, Mental Health Nursing Department of Nursing, Northumbria University, Newcastle-Upon-Tyne, United Kingdom; 6 Adjunct Professor Western Sydney University, Parramatta, NSW, Australia; Qatar University College of Nursing, QATAR

## Abstract

The outbreak of SARS-CoV-2, which causes COVID-19, has significantly impacted the psychological and physical health of a wide range of individuals, including healthcare professionals (HCPs). This umbrella review aims provide a quantitative summary of meta-analyses that have investigated the prevalence of stress, anxiety, depression, and sleep disturbance among HCPs during the COVID-19 pandemic. An umbrella review of systematic reviews and meta-analyses reviews was conducted. The search was performed using the EMBASE, PubMed, CINAHL, MEDLINE, PsycINFO, and Google Scholar databases from 01^st^ January 2020 to 15^th^ January 2024. A random-effects model was then used to estimate prevalence with a 95% confidence interval. Subgroup analysis and sensitivity analyses were then conducted to explore the heterogeneity of the sample. Seventy-two meta-analyses involved 2,308 primary studies were included after a full-text review. The umbrella review revealed that the pooled prevalence of stress, anxiety, depression, and sleep disturbance among HCPs during the COVID-19 pandemic was 37% (95% CI 32.87–41.22), 31.8% (95% CI 29.2–34.61) 29.4% (95% CI 27.13–31.84) 36.9% (95% CI 33.78–40.05) respectively. In subgroup analyses the prevalence of anxiety and depression was higher among nurses than among physicians. Evidence from this umbrella review suggested that a significant proportion of HCPs experienced stress, anxiety, depression, and sleep disturbance during the COVID-19 pandemic. This information will support authorities when implementing specific interventions that address mental health problems among HCPs during future pandemics or any other health crises. Such interventions may include the provision of mental health support services, such as counseling and peer support programs, as well as the implementation of organizational strategies to reduce workplace stressors.

## 1. Introduction

In December 2019, the coronavirus disease 2019 (COVID-19) pandemic emerged in Wuhan, China. The disease quickly spread worldwide, and the WHO declared a global health emergency in March 2020 [1]. Due to the COVID-19 pandemic, many countries implemented various measures to prevent the spread of the disease. These included implementing a partial or complete lockdown and social distancing strategies of varying intensity. The measures taken by these countries also affected the livelihood of individuals, an occurrence which might directly or indirectly also increase psychological morbidities. Undoubtedly, pandemics have a long history of impacting physical and mental health for different population groups, and HCPs are typically the most affected group in terms of bearing the burden of these illnesses [2]. In addition, several researchers have shown that work-related psychological disorders, including stress, anxiety, depression, and burnout, had already negatively affected the healthcare system before the COVID-19 pandemic, leading to low-quality care and high malpractice litigation [3–5].

As a result of the pandemic, HCPs experienced various changes in their personal and professional lives. For some, these included being given more responsibility, having to re-learn how to effectively control the infection, and dealing with the emotional impact of caring for infected and dying COVID-19 patients [6]. The alteration in their work environment, as well as the likelihood that they might acquire the infection themselves, can also affect their personal mental health. It is almost inevitable that the experiences of HCPs went through during the pandemic put them at heightened risk of stress, anxiety, depression, and sleep disturbance [7,8]. It is important to understand the effects of the pandemic on the mental health and well-being of HCPs in order to help plan strategies to prevent these individuals from experiencing detrimental effects, and to ensure that they can continue to deliver healthcare services.

During previous viral outbreaks including the Severe Acute Respiratory Syndrome (SARS) and the Middle East Respiratory Syndrome (MERS) epidemics, HCPs were placed under extraordinary amounts of pressure [9,10]. Indeed, evidence suggested that HCPs suffered from high levels of stress, anxiety, depression and sleep disturbance during these outbreaks [11,12]. A high prevalence of mental health problems can adversely impact the quality of life of HCPs, increase disability, turnover, absenteeism, and errors, and can deleteriously affect patient outcomes which may lead to low patient satisfaction [13]. Further, it might increase suicidal ideation or self-harming among HCPs [14].

In the present review, four phenomena were addressed. Sleep disturbance refers to a range of sleep-related problems, including disruptions in the body’s natural sleep-wake cycle, insufficient or poor-quality sleep, and sleep disorders [15]. The anxiety symptoms were defined as a state of excessive fear that translates to behavioural disturbances [16]. Major depressive disorder is a set of symptoms that includes depressed mood, loss of pleasure or interest, fatigue, changes in sleep and activity levels, and other symptoms, with a minimum duration of two weeks and at least five or more symptoms present according to the Diagnostic and Statistical Manual of Mental Disorders (DSM-5) [16]. Cohen et al., [17] define stress as “the degree to which individuals appraise situations in their lives as stressful”. In this umbrella review, stress, anxiety, depression, and sleep disturbance symptoms were defined based on the validated scales/questionnaires that assess each phenomenon in the original studies.

Several primary studies and, subsequently, systematic reviews and meta-analyses have been carried out to identify the prevalence of mental health problems among HCPs during the COVID-19 pandemic. Additionally, three umbrella reviews of meta-analyses [18–20] have been published previously, but the number of meta-analyses included in both cases did not exceed twenty. Since their publication, further meta-analyses have estimated the prevalence of stress, anxiety, depression, and sleep disturbance during the COVID-19 pandemic. The advantages of umbrella reviews include their ability to provide a comprehensive analysis of the literature, in this case about the prevalence of various mental disorders in HCPs during the COVID-19 pandemic. In addition, the results can then be used to make policy-level decisions to improve the quality of clinical care in terms of making clinical risk predictions and can inform future research priorities. Therefore, the aim of this umbrella review is to quantify meta-analytic findings aimed at estimating the prevalence symptoms of stress, anxiety, depression, and sleep disturbance among HCPs during the COVID-19 pandemic.

## 2. Methods

The umbrella review and meta-analysis were carried out according to the Preferred Reporting Items for Systematic Reviews and Meta-analyses (PRISMA) guidelines [21]. The review protocol was registered with the International Prospective Register of Systematic Reviews (PROSPERO) database and can be accessed online (CRD42022364721).

### 2.1 Search strategy

A systematic search was conducted to identify relevant meta-analyses in various electronic databases published between 1^st^ January 2020 and 15^th^ January 2024. The databases searched were PubMed, CINAHL, MEDLINE, EMBASE, PsycINFO, and Google Scholar. The search terms strategy used Medical Subject Headings (MeSH) and free text words with Boolean operators and truncations (AND/OR/NOT). The key search terms included (MH "Coronavirus Infections+") OR "COVID-19” OR "COVID” OR "coronavir* OR "Coronavirus" OR "SARS-COV2" AND "Health care provider" OR "health care professional" OR "healthcare provider*" OR (MH "Nurses+") OR (MH "Medical Staff") OR (MH "Physician") OR (MH "Medical Doctor") OR (MH "Staff Nurses") OR "nursing staff" OR "health personnel or health professional or nurse" OR "health personnel or health professional or nurse" AND "Stress" OR "post-traumatic stress disorder" OR "panic disorder" OR "obsessive compulsive disorder OR "anxi*" OR (MH "Anxiety Disorders+") OR (MH "Anxiety+") OR (MH "Depression+") OR "depress*" OR (MH "Affective Symptoms+") OR (MH "Affective Disorders+") OR (MH "Bipolar Disorder+") OR "affective" OR "mood" OR "mental" OR (MH "Mental Disorders+") OR (MH "Mental OR "psycho*" OR (MH "Insomnia+") OR (MH "Circadian Rhythm+") OR (MH "Sleep Disorders+") OR (MH "Insomnia+") OR (MH "Sleep+") AND "Systematic Review" OR "Meta-Analysis" OR "Meta-Analytic". Additionally, the reference lists were searched to find any other studies.

### 2.2 Study selection

Two reviewers (A.M.; A.A.) independently extracted the data from the search, scrutinizing all titles and abstracts for eligibility against the inclusion and exclusion criteria. A third reviewer (G.D.) was available to resolve any disagreements through discussion. Systematic reviews incorporating meta-analyses were included according to the following criteria. The studies: (1) examined the prevalence of stress or anxiety or depression or sleep disturbance symptoms; (2) presented results for HCPs as a group or separately (e.g., nurses or physicians only); further, studies involving non-HCPs must have presented results for HCPs separately and not pooled with non-HCPs.; (3) were conducted during the COVID-19 pandemic; (4) were published in English; (5) involved a systematic review with meta-analysis. Studies were excluded if (1) these consisted of a systematic review without meta-analysis; (2) consisted of a literature review or a narrative review (3) the participants were general population or non-HCPs.

### 2.3 Quality assessment

The methodological quality assessment of each meta-analysis was blindly rated by two reviewers using the Assessment of Multiple Systematic Reviews (AMSTAR-2) tool [22]. This scale consists of 16 items that evaluate the risk of bias of a systematic review. Items 1, 3, 5, 6, 10, 13, 14, and 16 are evaluated with either a "Yes" or "No" response. Items 2, 4, 7, 8, and 9 are evaluated with "Yes," "Partial Yes," or "No" responses. Items 11, 12, and 15 are evaluated with "Yes," "No," or "No meta-analysis conducted" responses. The overall rating can be rated as "High," "Moderate," "Low," or "Critically low."

### 2.4 Credibility of evidence

The credibility of the evidence of each association provided was evaluated by the Fusar-Poli and Radua [23] classification criteria. The level of evidence as convincing (class I) when specific criteria were met, including more than 1000 cases, p<10^−6^, I^2^ higher than 50%, 95% prediction intervals excluding the null, no small-study effects, and no publication bias. If the number of cases exceeded 1000, p<10^−6^, the largest study showed a statistically significant effect, but not all class I criteria were satisfied; the evidence level was considered highly suggestive (class II). When there were over 1000 cases, p<10^−3^, but no other class I or II criteria were met, the evidence level was termed suggestive (class III). If no class I-III criteria were met, the evidence level was classified as weak (class IV). The fourth level, termed weak evidence (class IV), included associations with a p ≤0.05, but these associations did not meet the criteria for class I, class II, or class III. The fifth level, denoted as non-significant (NS), comprised associations with a p˃0.05.

### 2.5 Data analyses

There are two methods exist for deriving effect size estimates from existing meta-analyses. The first approach involves conducting a meta-analysis on the effect size estimates taken from individual studies included in multiple prior meta-analyses [24]. However, this method demands significant time and resources. Furthermore, it contradicts the primary purpose of an umbrella review because it requires return to the original studies.

The second approach employs a statistical technique to efficiently summarize data from previous meta-analyses without the need to go back to the individual studies. This method relies solely on the summary effect sizes and their associated variances provided in the original meta-analyses [25]. It calculates an overall effect size for the combined meta-analyses by computing a weighted average of the summary effect sizes, with the weights determined by the inverse of the variances [26]. This approach is similar to the methods used in meta-analyses of primary studies. Although the second approach (combining summary effect sizes) may not achieve the same level of precision as the first method (combining all individual studies), empirical tests have confirmed its ability to generate a statistically valid estimate for the overall effect size [27,28]. In this umbrella review, we employed the second approach, which entailed the utilization of aggregate data derived from the meta-analyses.

The analyses were conducted using R software, version 4.3.1 (R Foundation for Statistical Computing), with packages used ‘meta’ [29], ‘metafore’ [30] and ‘metaumbrella’[31]. Pooled estimates prevalence with 95% Confidence Intervals (CIs) was conducted using random effect models, and the results were reported on a forest plot. In addition, the I-squared (I^2^) test was used to assess the statistical heterogeneity of the included meta-analyses. A value of I^2^ < 25% was considered low, 25–50% moderate, and ˃ 50% high [32]. Subgroup analyses were performed when there were at least four meta-analyses per subgroup.

Publication bias was assessed using Egger’s test with a p < 0.10 indicates a statistically significant small-study effect [33]. Statistical significance was set at p<0.05. If publication bias was identified, trim and fill methods were used to adjust the publication bias [34]. A sensitivity analysis was conducted in which individual meta-analyses were systematically removed one at a time to assess how they affected the overall combined prevalence of the remaining meta-analyses [35], with the aim of clarifying the stability and reliability of the finding [36].

## 3. Result

**Fig 1:**
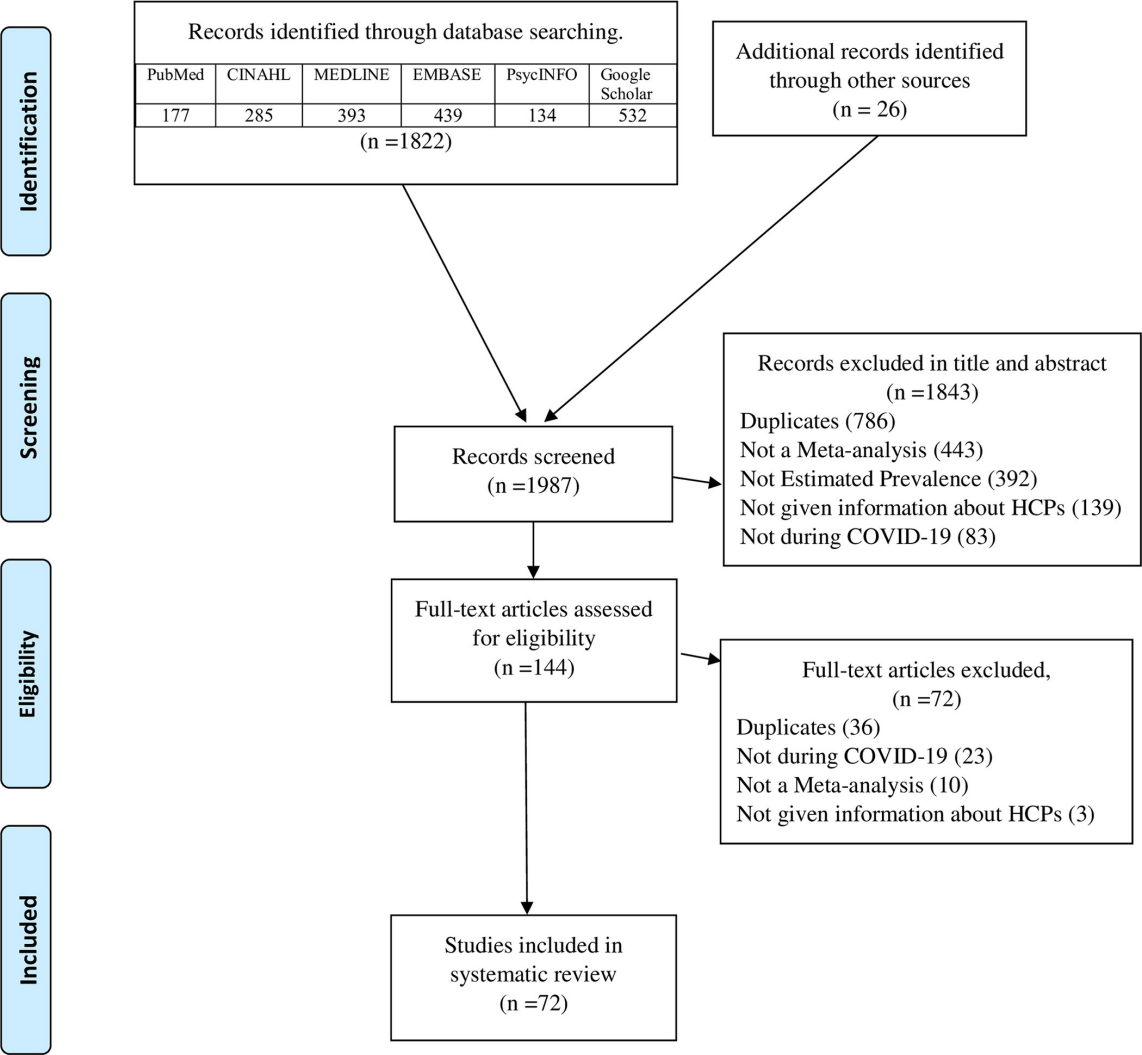
PRISMA diagram.

A total of 1,987 papers were identified through the database search. Out of these, 1,843 were excluded at the abstract and title screening stage for the following reasons: 786 were duplicates, 443 did not include a meta-analysis, 392 lacked information about prevalence, 139 lacked information about HCP status, and 83 were not conducted during the period of the COVID-19 pandemic. A further 72 papers were excluded during the full text review process. As a result, 72 meta-analyses were eligible for umbrella review (Fig 1).

### 3.1 General characteristics of the studies included

The included 72 meta-analyses [37–108]. Fifty-four of the meta-analyses dealt with HCPs in general, whereas two meta-analyses reported the situation only with regard to physicians or nurses [44,59], three meta-analyses dealt with nurses [49,79,91], and one dealt with physicians [88], while 17 meta-analyses included a mixed population (General and HCPs),(Only data specifically related to healthcare professionals were included in the umbrella review analysis). The most commonly used statistical software was STATA (n = 32), R (n = 17) and comprehensive meta-analysis (n = 11). Twenty-four meta-analyses used the Newcastle–Ottawa scale to assess the quality of the studies. Forty-six meta-analyses included mixed studies from different countries, twenty-five meta-analyses were conducted in specifical geographical areas: 10 for China, five for India, four for Asia two for Bangladesh and Ethiopia and one for each of the following: Egypt, South Asia, and Vietnam. The detailed characteristics of the studies including meta-analyses are shown in Table 1.

**Table 1:** Characteristics of the included meta-analyses (N = 72).

	Study	Total Study Included	Outcome	Population	Study Included	Prevalence	95%CI	I^2^	Events	Sample size	Analysis Software	Model	Appraisal Tool	AMSTAR-2 Quality	Remarks
1	(Batra et al. 2020) [38]	65	Stress	All	17	40.3	31.4–50.0	99.1	6543	16235	CMA	REM	NIH	Moderate	HCPs
			Anxiety	All	46	34.4	29.5–39.7	99.1	17749	51596					
				Nurses	8	39.3	27.5–52.5	98.9	8606	21899					
				Physicians	8	32.5	21.9–45.2	99.0	7117	21899					
			Depression	All	46	31.8	26.8–37.2	99.2	16906	53164					
				Nurses	9	42.4	30.4–5.4	99.0	9301	21936					
				Physicians	9	39.1	27.3–52.2	98.4	8577	21936					
2	(Krishnamoorthy et al. 2020) [39]	23	Stress	All	5	33	19–50	98.4	1986	6017	STATA	REM	NOS	Moderate	Mixed
			Anxiety	All	16	24	16–32	99.6	9201	38337					
			Depression	All	16	25	19–32	99.4	9425	37701					
			Sleep Disturbance	All	5	43	28–59	99.2	2010	4675					
3	(Pappa et al. 2020) [41]	13	Anxiety	All	12	23.2	17.8–29.1	99	7367	31756	MetaXL	REM	NOS	Moderate	HCPs
				Nurses	6	25.8	19.2–33.0	98	5160	19999					
				Physicians	6	21.7	15.3–29	97	4340	19999					
			Depression	All	10	22.8	15.1–31.5	99.6	7071	31014					
				Nurses	5	30.3	18.2–43.8	99.5	5679	18742					
				Physicians	5	25.4	16.6–35.2	98	4760	18742					
			Sleep Disturbance	All	5	34.3	27.5–41.5	98	2935	8558					
4	(Qiu et al. 2020) [42]	52	Sleep Disturbance	All	52	39.2	36.0–427	98.3	12446	31749	R	REM	Loney	Low	HCPs/ China
				Nurses	41	39.4	35.7–43.5	98.4	9878	25070					
				Physicians	3	34.2	12.6–55.8	99.2	720	2104					
5	(Salari et al. 2020a) [44]	7	Sleep Disturbance	Nurses	6	34.8	24.8–46.4	97.4	2526	7259	CMA	REM	STROBE	Critically Low	HCPs
				Physicians	5	41.6	27.7–57	97.3	2286	5496					
6	(Salari et al. 2020b) [45]	29	Stress	All	8	36.4	18.3–59.5	99	1293	3551	CMA	REM	STROBE	Critically Low	HCPs /Frontline
			Anxiety	All	17	27	20.1–35.3	95.5	2987	11062					
			Depression	All	15	20.6	13.1–30.9	99.1	2196	10658					
7	(Allan et al. 2020) [37]	9	Stress	All	9	23.4	16.3–31.2	96	970	4147	R	REM	NIH	Critically Low	HCPs
8	(Salazar de Pablo et al. 2020) [46]	15	Stress	All	3	29.9	8.9–65.2	99.7	2030	6789	CMA	REM	MMAT	Moderate	Mixed
			Anxiety	All	4	22.2	12.7–35.8	99	1713	7716					
			Depression	All	4	17.9	6.7–40.1	99.6	1381	7716					
			Sleep Disturbance	All	3	44.5	38.2–50.9	93	1553	3490					
9	(Ren et al. 2020) [43]	5	Anxiety	All	4	27	12–43	99.4	1308	4843	STATA	REM	AHRQ	Low	Mixed
			Depression	All	3	25	4–45	99.7	1166	4663					
10	(Pan et al. 2020) [40]	7	Anxiety	All	7	11.4	7.1–15.8	99	848	7441	STATA	REM	STROBE	Low	Mixed
11	(Zhang et al. 2021) [82]	26	Stress	All	8	42.1	26.0–60.0	99.5	3866	9183	R	REM	NIH	Moderate	HCPs/ China
			Anxiety	All	23	27.0	21.0–34.0	99.0	5791	21447					
			Depression	All	18	26.2	21.0–33.0	98.6	4930	18818					
			Sleep Disturbance	All	8	35.5	28.0–42.0	98.3	4174	11758					
12	(Adibi et al. 2021) [48]	19	Anxiety	All	19	30.5	25.6–35.4	98.4	6669	21866	STATA	REM	NG	Low	HCPs
13	(Al Maqbali et al. 2021) [49]	93	Stress	Nurses	40	43	37–49	98	11625	27034	CMA	REM	NOS	Moderate	Nurses
			Anxiety	Nurses	73	37	32–41	99	30178	81561					
			Depression	Nurses	62	35	31–39	99	26947	76992					
			Sleep Disturbance	Nurses	18	43	36–50	96	4600	10697					
14	(Alimoradi et al. 2021) [50]	62	Sleep Disturbance	All	62	43	39–47	99.3	25521	59350	STATA	REM	NOS	Low	Mixed
15	(Bareeqa et al. 2021) [51]	8	Depression	All	8	31.5	20.7–43.5	99.3	3234	10267	OMA	REM	NOS	Low	Mixed/China
16	(Cénat et al. 2021) [52]	27	Stress	All	4	21.0	9.0–43.0	99.7	1444	6878	R	REM	JBI	Moderate	Mixed
			Anxiety	All	23	15.9	12.2–20.3	99	5895	37076					
			Depression	All	18	14	11.0–17.0	95.3	4781	34151					
			Sleep Disturbance	All	6	16	8.0–30.0	99.8	993	6209					
17	(Ching et al. 2021) [53]	148	Stress	All	40	36.4	23.2	49.7	12380	34010	OMA	REM	STROBE	Low	HCPs/Asia
			Anxiety	All	117	39.7	34.3–45.1	99.8	39557	99639					
			Depression	All	98	37.5	33.8–41.3	99.5	38861	103628					
18	(Dutta et al. 2021) [57]	33	Stress	All	17	37.7	24.0–52.3	100	10269	27238	MetaXL	REM	NOS	Low	HCPs
			Anxiety	All	31	32.5	26.4–39	99	7628	23472					
			Depression	All	30	32.4	25.6–39.3	99	12200	37655					
			Sleep Disturbance	All	11	36.6	36.6–48.3	99	3810	10411					
19	(Hao et al. 2021) [60]	20	Stress	All	5	25.6	11.8–39.4	99	790	3085	R	REM	AHRQ	Low	HCPs
			Anxiety	All	16	28.6	22.4–36.4	99	2864	10015					
				Nurses	7	23.5	15.8–35	99	1437	6113					
			Depression	All	14	24.1	16.2–32.1	99	1915	7948					
				Nurses	5	25	12.7–37.4	96	423	1692					
			Sleep Disturbance	All	5	44.1	31.3–57	98	1607	3643					
20	(Yan et al. 2021) [81]	35	Stress	All	4	56	32–79	97	428	765	R	REM	STROBE	Low	HCPs /China
			Anxiety	All	29	41	35–47	98	6439	15704					
			Depression	All	20	27	20–32	94	5680	21038					
			Sleep Disturbance	All	8	41	33–50	98	3065	7476					
21	(Liu et al. 2021) [65]	33	Stress	All	5	30.6	9.1–65.9	98	979	3200	CMA	REM	NOS	Low	Mixed
			Anxiety	All	31	32.7	29.9–38.2	98	12340	37736					
			Depression	All	23	25.8	20.4–31.0	98	9347	36230					
			Sleep Disturbance	All	8	37.3	32.1–42.8	98	3649	9784					
22	(Mahmud et al. 2021) [66]	69	Stress	All	41	44.9	37–31.1	99.8	37170	82783	STATA	REM	STROBE	Low	HCPs
			Anxiety	All	75	41.4	36.2–46.7	99.8	61038	147435					
			Depression	All	69	37.1	31.8–42.4	99.8	53665	144649					
			Sleep Disturbance	All	21	43.8	35.8–51.7	99.4	14616	33370					
23	(Marvaldi et al. 2021) [67]	70	Stress	All	9	31.4	17.5–47.3	99.8	7979	25412	OMA	REM	AHRQ	Moderate	HCPs
			Anxiety	All	22	30	24.2–37	99.6	15583	51942					
			Depression	All	25	31.1	25.7–36.8	996	21157	68030					
			Sleep Disturbance	All	10	44	24.6–64.5	99.8	5468	12428					
24	(Phiri et al. 2021) [70]	83	Stress	All	19	23.2	10.5–35.9	99.8	10666	45976	STATA	REM	NOS	Moderate	Mixed
			Anxiety	All	67	21.9	18.7–25	99.7	24296	110940					
			Depression	All	67	23.4	20.6–26.3	99.5	28699	122644					
			Sleep Disturbance	All	17	24	16.4–31.6	99.5	9626	40110					
25	(Santabárbara et al. 2021) [73]	71	Anxiety	All	71	25	21–29	99.1	14641	58565	STATA	REM	JBI	Low	HCPs
				Nurses	17	27	20–34	97.8	1856	6875					
				Physicians	13	17	12–22	95.6	880	5177					
26	(Saragih et al. 2021) [77]	38	Stress	All	14	37	25–50	99.8	5695	15391	STATA	REM	JBI	Low	HCPs
			Anxiety	All	33	40	29–52	99.8	12453	31132					
			Depression	All	30	37	29–45	99.7	16738	45238					
27	(Singh et al. 2021) [76]	6	Stress	All	6	65.1	48.2–80.3	98.6	1577	2423	STATA	REM	NIH	Critically Low	Mixed/ India
			Anxiety	All	5	35.3	26.3–44.9	92.3	658	1863					
			Depression	All	6	35.4	25.1–46.4	97.6	1534	4333					
28	(Sun et al. 2021) [77]	47	Anxiety	All	44	37	31–42	99.9	23828	64401	STATA	REM	NOS	Low	HCPs
				Nurses	9	34	26–41	99	9310	27382					
				Physicians	9	28	19–38	99.1	7667	27382					
			Depression	All	39	39	31–41	99.6	30395	77936					
				Nurses	8	38	30–46	99	10339	27209					
				Physicians	8	33	26–40	97.9	8979	27209					
			Sleep Disturbance	All	9	32	23–42	99.5	4040	12626					
29	(Varghese et al. 2021) [79]	26	Stress	Nurses	10	40.6	25.6–56.8	98.6	1707	4204	OMA	REM	Loney	Low	Nurses
			Anxiety	Nurses	21	33	24–43	99.4	4502	13641					
			Depression	Nurses	17	32	21–44	99.4	3934	12294					
30	(Wu et al. 2021) [95]	29	Stress	All	5	41.2	19.8–64.5	99.8	4188	10165	STATA	REM	STROBE	Moderate	Mixed
			Anxiety	All	23	29	23.6–34.7	99.4	14541	50143					
			Depression	All	23	31	24.7–37.5	99.5	12986	41889					
			Sleep Disturbance	All	7	47.3	38.8–55.8	98.7	6326	13375					
31	(Xia et al. 2021) [80]	17	Sleep Disturbance	All	15	45.1	37.2–53.1	98.7	5530	12261	STATA	REM	Loney	Moderate	HCPs /China
32	(Deng et al. 2021) [55]	34	Anxiety	All	22	40	33–46	99	4560	11401	R	REM	AHRQ	Low	HCPs /China
			Depression	All	20	31	25–37	98.4	3546	11438					
33	(Dong et al. 2021) [56]	22	Stress	All	9	29.1	24.3–33.8	96.9	4258	14631	STATA	REM	AHRQ	Moderate	HCPs /China
			Anxiety	All	22	34.4	29.5–39.4	98.8	10338	30052					
			Depression	All	18	31.1	24.5–37.7	99.2	6827	21953					
34	(Serrano-Ripoll et al. 2021) [75]	13	Sleep Disturbance	All	13	38	37–39	99	5349	14075	STATA	REM	MU	Moderate	HCPs
35	(Hossain et al. 2021) [62]	17	Anxiety	All	15	43.6	33.1–54.5	99.2	6557	15038	STATA	REM	NOS	Low	Mixed/South Asia
			Depression	All	14	29.9	23.9–36.6	98.1	4984	16670					
36	(Li et al. 2021) [64]	65	Stress	All	9	21.5	10.5–34.9	99.7	5254	24439	STATA	REM	MU	Low	HCPs
			Anxiety	All	57	22.1	18.2–26.3	99.4	16416	74280					
			Depression	All	55	21.7	18.3–25.2	99.3	18373	84666					
37	(El-Qushayri et al. 2021) [58]	10	Stress	All	6	66.6	47.6–81.3	98	1273	1911	CMA	REM	NIH	Moderate	HCPs/Egypt
			Anxiety	All	4	71.9	49.4–86.9	98	966	1344					
			Depression	All	5	65.5	46.9–80.3	98	1090	1664					
38	(Jahrami et al. 2021) [63]	11	Sleep Disturbance	All	11	36	21.1–54.2	99	1747	4854	R	REM	NOS	Moderate	Mixed
39	(Olaya et al. 2021) [69]	57	Depression	All	46	24	20–25	99.3	12846	53527	STATA	REM	JBI	Low	HCPs
				Nurses	14	25	18–33	97.7	1471	5883					
				Physicians	10	24	16–31	96.9	1024	4266					
40	(Raoofi et al. 2021) [71]	46	Anxiety	All	46	26.1	19–34.6	99	16065	61551	R	REM	NOS	Low	HCPs
				Nurses	31	24.7	17.6–33.5	99	3074	12447					
				Physicians	17	24	12.6–40.9	99	1239	5162					
41	(Salehi et al. 2021) [72]	4	Stress	All	4	11	5–16	95	217	1977	STATA	REM	STROBE	Moderate	Mixed
42	(Abdulla et al. 2021) [47]	23	Stress	All	12	58.1	44.8–71.3	99	2445	4209	Rev	REM	27-itmes	Moderate	HCPs /India
			Anxiety	All	10	42.9	30.3–55.5	98	1312	3059					
			Depression	All	5	41.9	29.2–54.6	99	2429	5796					
43	(Crocamo et al. 2021) [54]	14	Depression	All	14	23.8	16.2–32.2	99	3373	14173	STATA	REM	NOS	Low	HCPs
44	(Halemani et al. 2021) [59]	13	Stress	Nurses	4	37	8–66	99.6	720	1946	STATA	REM	NOS	Low	HCPs
				Physicians	3	37	6–68	99	343	928					
			Anxiety	Nurses	13	42	33–50	97	1750	4167					
				Physicians	12	34	26–42	95	1127	3315					
			Depression	Nurses	13	42	32–50	97	1750	4167					
				Physicians	12	34	23–45	97	1127	3315					
			Sleep Disturbance	Nurses	4	44	35–53	91	637	1447					
				Physicians	4	35	26–43	91	500	1428					
45	(Hosen et al. 2021) [61]	4	Anxiety	All	4	52	32–71	96	883	1699	STATA	REM	JBI	Low	Mixed/ Bangladesh
			Depression	All	4	41	35–46	62.1	697	1699					
46	(Norhayati et al. 2021) [68]	80	Stress	All	20	31.7	21.3–42.2	98	4017	12673	Rev	REM	JBI	Low	HCPs/Asia
			Anxiety	All	68	34.8	30.1–38.8	100	43474	124925					
				Nurses	18	36.1	23.8–48.4	100	4178	11574					
				Physicians	18	30.1	20.6–39.6	99	2191	7279					
			Depression	All	60	34.6	30.9–38.4	100	45779	132308					
				Nurses	19	36.6	27.1–46.2	99	3718	10159					
				Physicians	19	28.3	18.9–37.8	99	1937	6845					
			Sleep Disturbance	All	12	37.9	25.4–50.4	100	5638	14877					
47	(Zhao et al. 2021) [83]		Stress	All	5	28	9.5–59	99	1212	4327	CMA	REM	Loney	Low	Mixed
			Anxiety	All	14	23.	17.1–30.8	98	2995	13020					
			Depression	All	11	23.9	15–35.9	99	2849	11922					
48	(Thakur and Pathak 2021) [78]	20	Stress	All	3	55.6	36–110	99	1356	2438		REM	NOS	Low	HCPs /Frontline
			Anxiety	All	10	27.2	18.1–36.3	98	3229	11873					
			Depression	All	9	32	18–46	99	3604	11262					
			Sleep Disturbance	All	3	34.4	32.5–36.3	99	1296	3768					
49	(Aymerich et al. 2022) [84]	239	Stress	All	57	40	32–47	99.87	19217	48042	STATA	REM	NOS	Low	HCPs
			Anxiety	All	176	42	35–48	99.94	86735	206513					
			Depression	All	160	33	28–38	99.95	69551	210762					
			Sleep Disturbance	All	55	42	36–48	99.57	15569	37068					
50	(Hu et al. 2022) [86]	71	Anxiety	All	60	26.7	19.6–34.6	100	20558	76998	R	REM	JBI	Low	HCPs/China
			Depression	All	56	28.7	19.6–34.6	100	25655	89390					
			Sleep Disturbance	All	25	40.4	33.8–47.2	99	10883	26937					
51	(Johns et al. 2022) [88]	55	Anxiety	Physicians	30	25.8	20.4–31.5	99.2	8586	33281	R	REM	JBI	Low	Physicians
			Depression	Physicians	26	20.5	16–25.3	98.9	6447	31447					
52	(Li et al. 2022) [89]	48	Sleep Disturbance	All	48	46.4	40.3–52.5	99.5	15322	33021	STATA	REM	AHRQ	Low	Mixed/China
53	(Rezaei et al. 2022) [90]	24	Depression	All	24	26	18–35		10923	42010	R	REM	NOS	Low	HCPs
54	(Xiong et al. 2022) [93]	44	Stress	All	7	27	16–38	99.8	7330	27148	STATA	REM	AHRQ	Low	HCPs /China
			Anxiety	All	18	17	13–21	99.5	5915	34793					
			Depression	All	15	15	13–16	94.3	7293	48621					
			Sleep Disturbance	All	5	15	7–23	99	857	5711					
55	(Zhang et al. 2022) [94]	88	Anxiety	All	88	33.6	29.7–37.6	99.2	25222	75066	STATA	REM	STROBE	Low	HCPs
56	(Ślusarska et al. 2022) [91]	23	Anxiety	Nurses	22	29	18–40	99.9	12488	43062	R	REM	AHRQ	Low	HCPs
			Depression	Nurses	18	22	15–30	99.7	8675	39430					
57	(Blasco-Belled et al. 2022) [85]	74	Stress	All	24	32	23–42	100	8122	25382	MetaXL	REM	JBI	Moderate	Mixed
			Anxiety	All	66	31	27–36	99	22309	71966					
			Depression	All	60	31	26–35	99	26269	84740					
58	(Huang et al. 2022) [87]	17	Stress	All	3	17	4–34	85	45	265	CMA	REM	Hoy’s	Low	HCPs/Frontline
			Anxiety	All	16	32	20–44	98	2494	7795					
			Depression	All	14	31	21–41	97	2326	7504					
59	(Tran et al. 2022) [92]	7	Depression	All	7	17.3	9.2–27.3	100	1555	8987	CMA	REM	JBI	Low	HCPs /Vietnam
60	(Tong et al. 2022) [105]	19	Stress	All	10	53	41.1–64.9	78.3	3167	5976	R	REM	NOS	Moderate	HCPs
			Anxiety	All	19	43	33.8–52.3	99	7316	17013					
			Depression	All	19	44.6	36.1–53.1	99	7588	17013					
			Sleep Disturbance	All	16	42.9	33.9–51.9	99	6612	15413					
61	(Andhavarapu et al. 2022) [96]	119	Stress	All	119	34	30–39	90	39829	117143	CMA	REM	NOS	Moderate	HCPs
62	(Mamun et al. 2022) [103]	4	Sleep Disturbance	All	4	51	23–79	98	575	1127	STATA	REM	JBI	Moderate	HCPs/Bangladesh
63	(Cheung et al. 2022) [98]	6	Anxiety	All	6	37.8	28.7–46.9	96	1230	3253	STATA	REM	JBI	Moderate	HCPs/Asia
			Depression	All	6	39.8	29–50.5	97	1295	3253					
64	(Hasen et al. 2023b) [100]	8	Depression	All	7	40	23–57	99	1101	2752	STATA	REM	NOS	Moderate	HCPs/Ethiopia
			Sleep Disturbance	All	3	37	13–58	98	334	904					
65	(Athe et al. 2023) [97]	11	Stress	All	6	50.4	22.6–78.2	99	585	1161	RevMan	REM		Critically Low	HCPs/India
			Anxiety	All	10	42.9	30.3–55.5	98	1302	3036					
			Depression	All	8	35.4	24.5–46.3	97	1114	3147					
66	(Sialakis et al. 2023) [107]	14	Anxiety	All	14	41.4	30.3–52.9	99	3221	7780	MedCalc	REM	JBI	Moderate	HCPs
			Depression	All	14	33.8	24.7–43.6	98	2630	7780					
67	(Gheshlagh et al. 2023) [99]	12	Anxiety	All	12	23	18–27	98	6891	29960	STATA	REM	NOS	Moderate	HCPs/ Asia
			Depression	All	11	20	14–27	99	5890	29448					
68	(Wang et al. 2023) [106]	14	Stress	Nurses	14	65.4	55.9–79.9	99	13902	21257	STATA	REM	NOS	Moderate	HCPs
69	(Khobragade and Agrawal 2023) [101]	39	Stress	All	23	43	30–56	99	3494	8125	R	REM	NOS	Critically Low	HCPs/India
			Sleep Disturbance	All	16	35	28–44	97	1741	4974					
70	(Lee et al. 2023) [102]		Stress	All	107	25.5	22.5–28.6	100	47070	184588	R	REM	JBI	Moderate	HCPs
				Nurses	42	27.4	22.5–32.5	99	10016	36554					
				Physicians	42	22.4	16.4–29.1	99	5635	25155					
			Anxiety	All	272	28.7	26.5–31	99	84831	295578					
				Nurses	85	31.5	26.9–36.3	99	22899	72695					
				Physicians	81	26.9	23–31	98	10072	37443					
			Depression	All	274	28.5	26.5–30.7	99	94019	329891					
				Nurses	98	28	24.5–31.7	99	24327	86881					
				Physicians	89	25.3	21.8–29	99	10417	41175					
			Sleep Disturbance	All	54	24.4	19.4–29.9	99	7536	30886					
				Nurses	14	26	16–37.3	99	1644	6324					
				Physicians	15	16	10.2–22.7	95	632	3948					
71	(Sharma et al. 2023) [104]		Stress	All	22	36	23.7–48.2	99	2875	7985	R	OMA	JBI	Moderate	HCPs/India
			Anxiety	All	20	25	18.4–31.6	98	1953	7811					
			Depression	All	21	20.1	15.6–24.6	97	2055	10222					
			Sleep Disturbance	All	6	18.9	9.9–28	95	296	1565					
72	(Hasen et al. 2023a) [108]	13	Stress	All	9	51	41–62	98	1929	3815	STATA	REM	NOS	Moderate	HCPs/Ethiopia
			Anxiety	All	8	46	30–61	99	1802	3703					

National Institutes of Health: NIH; Joanna Briggs Institute (JBI); OMA: Open Meta Analyst;GRADE: Grading of Recommendations Assessment, Development and Evaluations; NOS: Newcastle–Ottawa Scale; AHRQ: Agency for Healthcare Research and Quality; MMAT: Mixed Methods Appraisal Tool; MU: McMaster University; SAQOR: Systematic Assessment of Quality in Observation Research; CMA: Comprehensive Meta-Analysis software; HCPs: Healthcare Professionals.

### 3.2 Quality appraisal

Each meta-analysis was assessed using the AMSTAR-2 tool. Twenty- nine meta-analyses were classified as moderate quality and thirty-seven as low quality. Only six meta-analyses were classified as critically low quality [37,44,45,76,97,101].

### 3.3 Prevalence of stress

**Fig 2:**
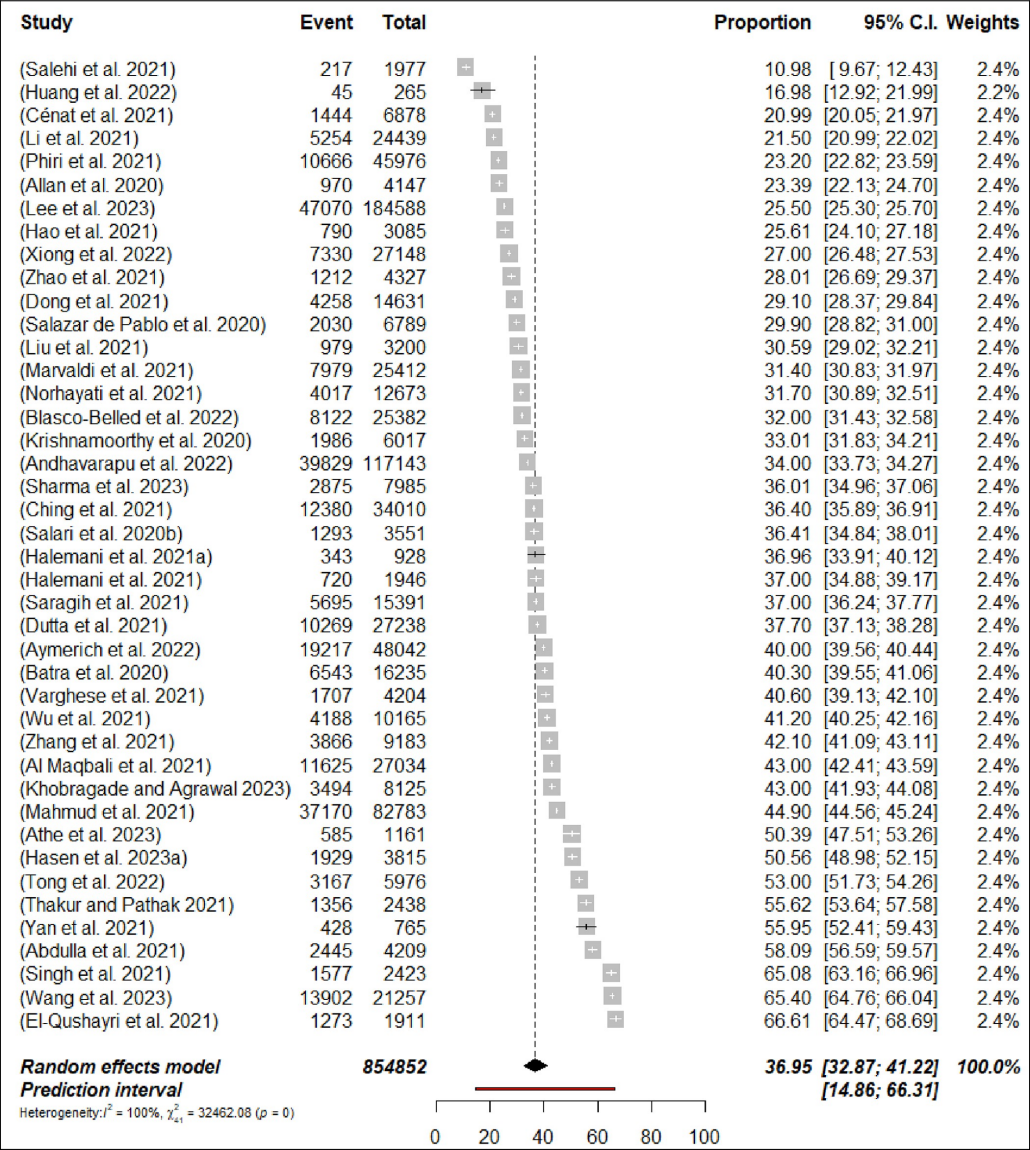
Forest plot of the prevalence of stress among HCPs stress (N = 42).

**Fig 3:**
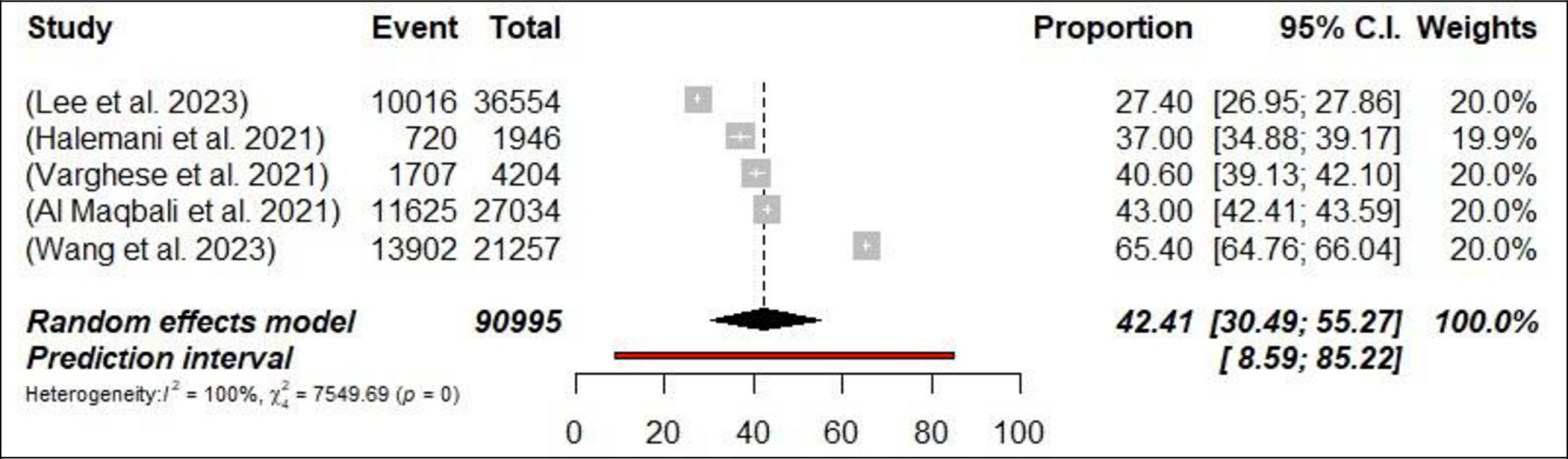
Forest plot of the prevalence of stress among nurses (N = 5).

Stress was reported in 42 meta-analyses among HCPs. The estimation of prevalence for stress varied between 11% [72] and 66.6% [58] (Fig 2: Forest Plots). The pooled prevalence of stress from was 37% (292,245/854,852 participants, 95% CI 32.87–41.22) with 95% PI: 14.86–66.3. There was significant heterogeneity between meta-analyses when it came to estimating the prevalence of stress (*p*< 0.0001, I^2^ = 99.9%). In the subgroup analysis, the prevalence of anxiety among nurses was determined to be 42.6% (n = 5; 95% CI = 30.49–55.27, I^2^ = 99%), as shown in Fig 3: Forest Plots. However, the analysis for physicians was not conducted due to an insufficient number of available meta-analyses. In sensitivity analysis, none of the meta-analyses resulted in changes to the pooled prevalence estimates greater than a 2%. The prevalence rate estimates for stress were considered to be suggestive evidence (class III) (Seen Table 2).

**Table 2:** Level of evidence for the prevalence of symptoms among HCPs.

	No	Total	Event	Prevalence %	Random effects (95% CI)	Random effects p-value	95% Prediction Interval	I^2^, %	Egger Test	Begg	Class of Evidence	Sensitivity Analysis
**Stress**												
HCPs	42	854852	292245	37	32.87–41.22	<0.0001	14.86–66.3	99.9	0.19	0.79	III	± 2%
Nurses	5	90995	37970	42.4	30.49–55.27	<0.0001	8.59–85.22	99.9	0.87	1	III	± 3%
**Anxiety**												
HCPs	55	2310774	734036	31.8	29.2–34.61	<0.0001	15.24–54.83	99.9	0.23	0.53	III	± 1%
Nurses	12	321415	105438	31.6	28.33–35.14	<0.0001	19.54–46.86	99.6	0.31	0.58	III	± 1%
Physician	9	160937	43219	26.3	22.89–30.10	<0.0001	14.98–42.05	99.2	0.65	1	III	± 1%
**Depression**												
HCPs	54	2349613	698808	29.4	27.13–31.84	<0.0001	15.01–49.62	99.9	0.16	0.84	III	± 0.5%
Nurses	11	305385	96564	32	28–36.35	<0.0001	17.98–50.34	99.8	0.82	0.94	III	± 1%
Physician	8	154935	43268	28.4	24.32–32.78	<0.0001	15.37–46.32	99.8	0.98	0.62	III	± 1%
**Sleep**												
HCPs	36	502780	191673	36.9	33.78–40.05	<0.0001	19.99–57.70	99.7	0.24	0.04	III	± 1%
Nurses	5	50797	19285	37.1	30.71–44.1	<0.0001	30.71–44.06	99.3	0.33	0.61	III	± 2%
Physician	4	12976	4138	30.6	20.04–43.77	<0.0001	2.68-87-62	99.5	0.48	1	III	± 3%

### 3.4 Prevalence of anxiety

**Fig 4:**
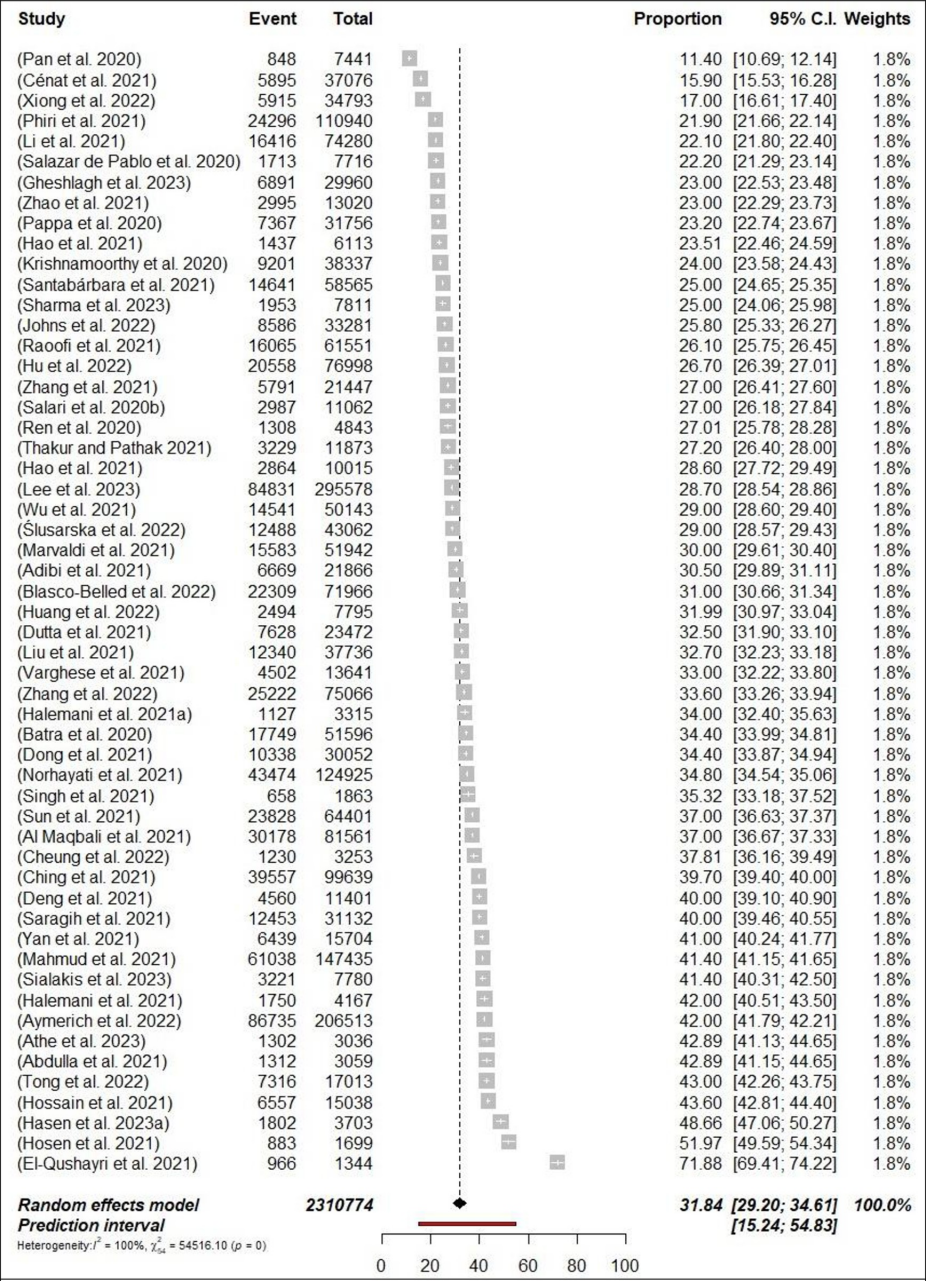
Forest plot of the prevalence of anxiety among HCPs (N = 55).

**Fig 5:**
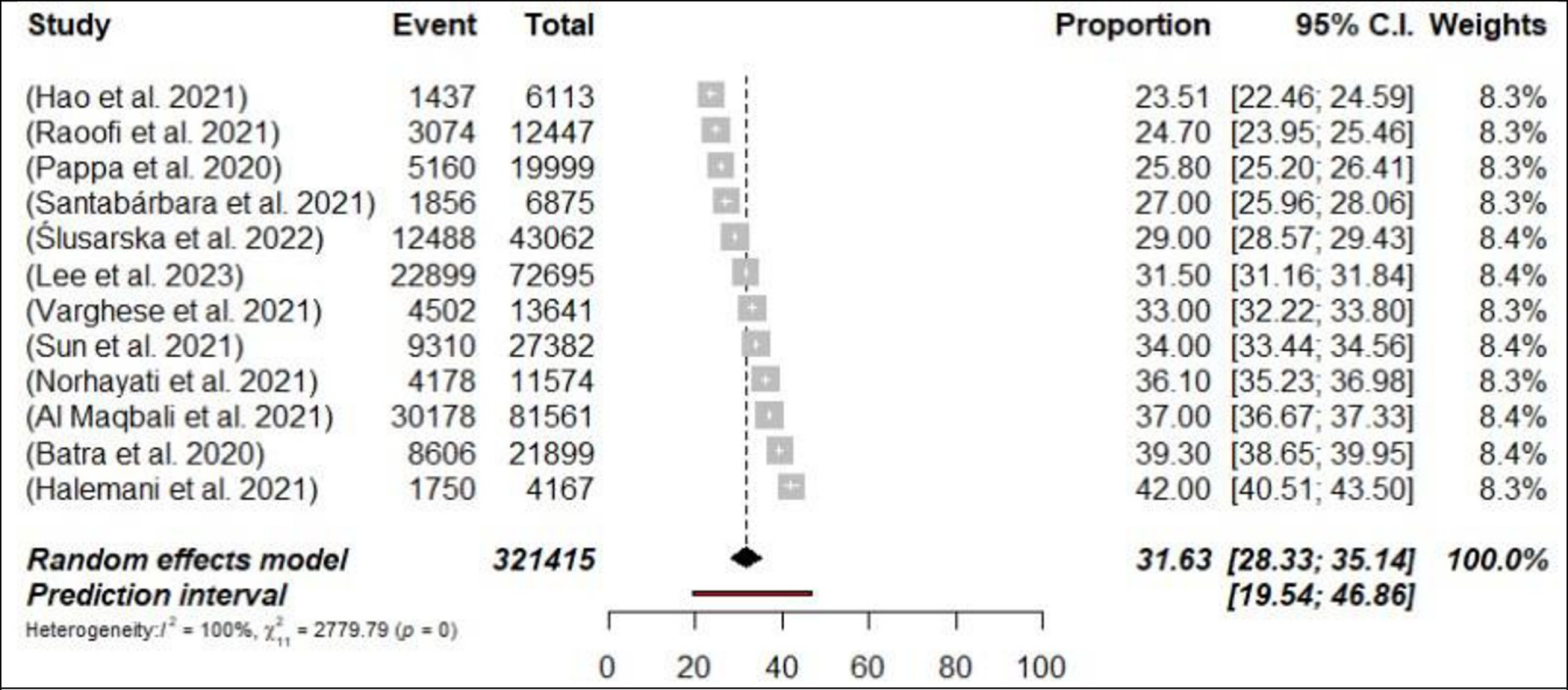
Forest plot of the prevalence of anxiety among nurses (N = 12).

**Fig 6:**
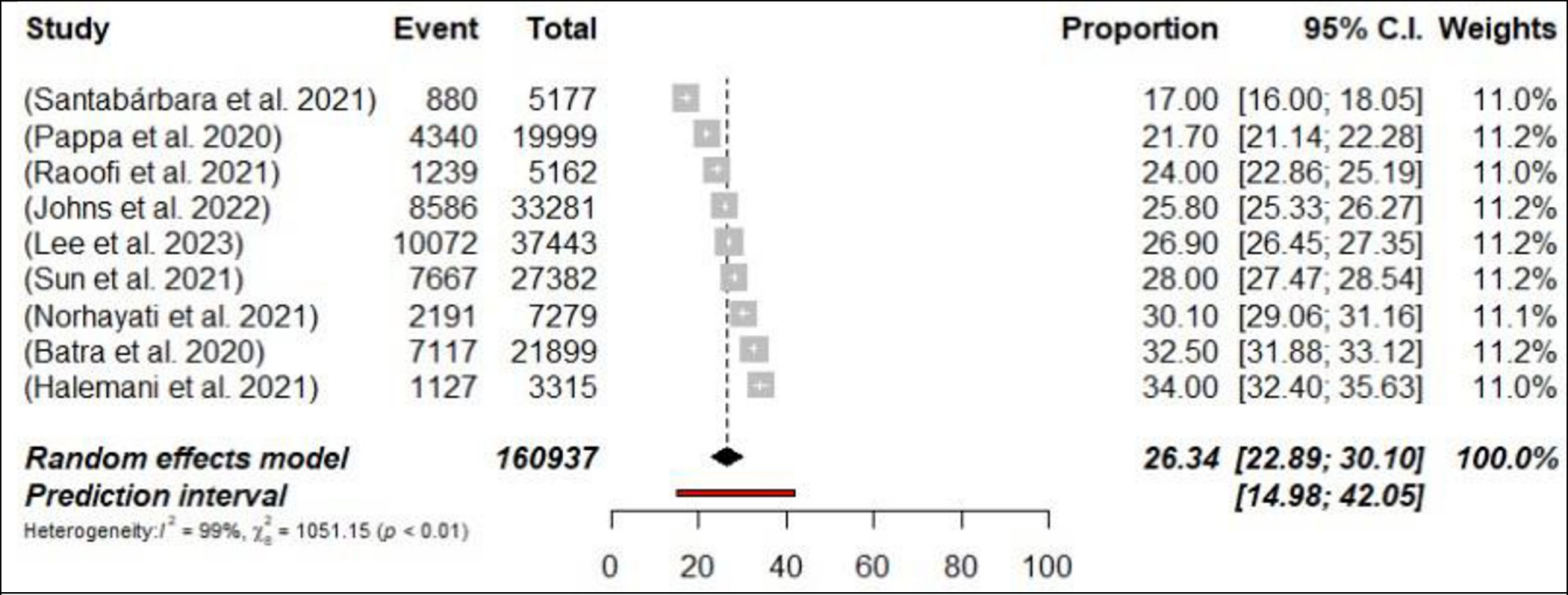
Forest plot of the prevalence of anxiety among physicians (N = 9).

Fifty-five meta-analyses estimated the prevalence of anxiety among HCPs, and ranged from 11.4% [40] to 71.9% [58]. The pooled prevalence of anxiety was 31.8% (734,036/2,310,774 participants, 95% CI 29.2–34.61) with 95% PI: 15.24–54.83 (Fig 4: Forest Plots) among all HCPs and there was considerable heterogeneity (*p*< 0.0001, I^2^ = 99.9%). In the subgroup analyses, in terms of professional status, the pooled prevalence of anxiety was 31.6% (n = 12; 95% CI = 28.33–35.14, I^2^ = 99%) and 26.3% (n = 9; 95% CI = 22.89–30.10, I^2^ = 99%) for nurses, and physicians respectively (Figs 5 and 6: Forest Plots). A sensitivity analysis, specifically a leave-one-out analysis, revealed that none of the meta-analyses had an impact on the global prevalence estimate of anxiety symptoms greater than 1%. Suggestive evidence (class III) was found for the estimated prevalence of anxiety in the case of HCPs, nurses and physicians.

### 3.5 Prevalence of depression

**Fig 7:**
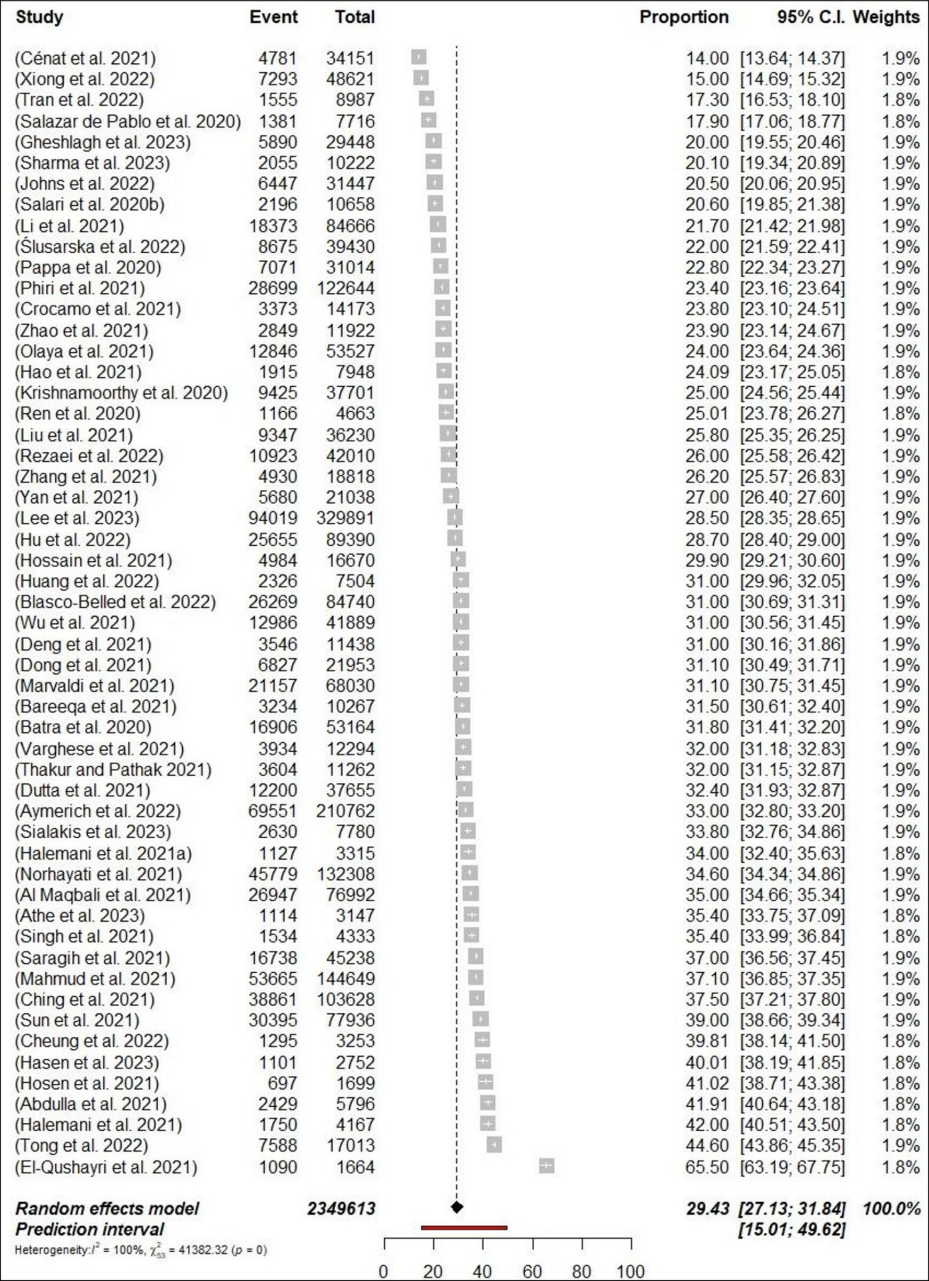
Forest plot of the prevalence of depression among HCPs (N = 54).

**Fig 8:**
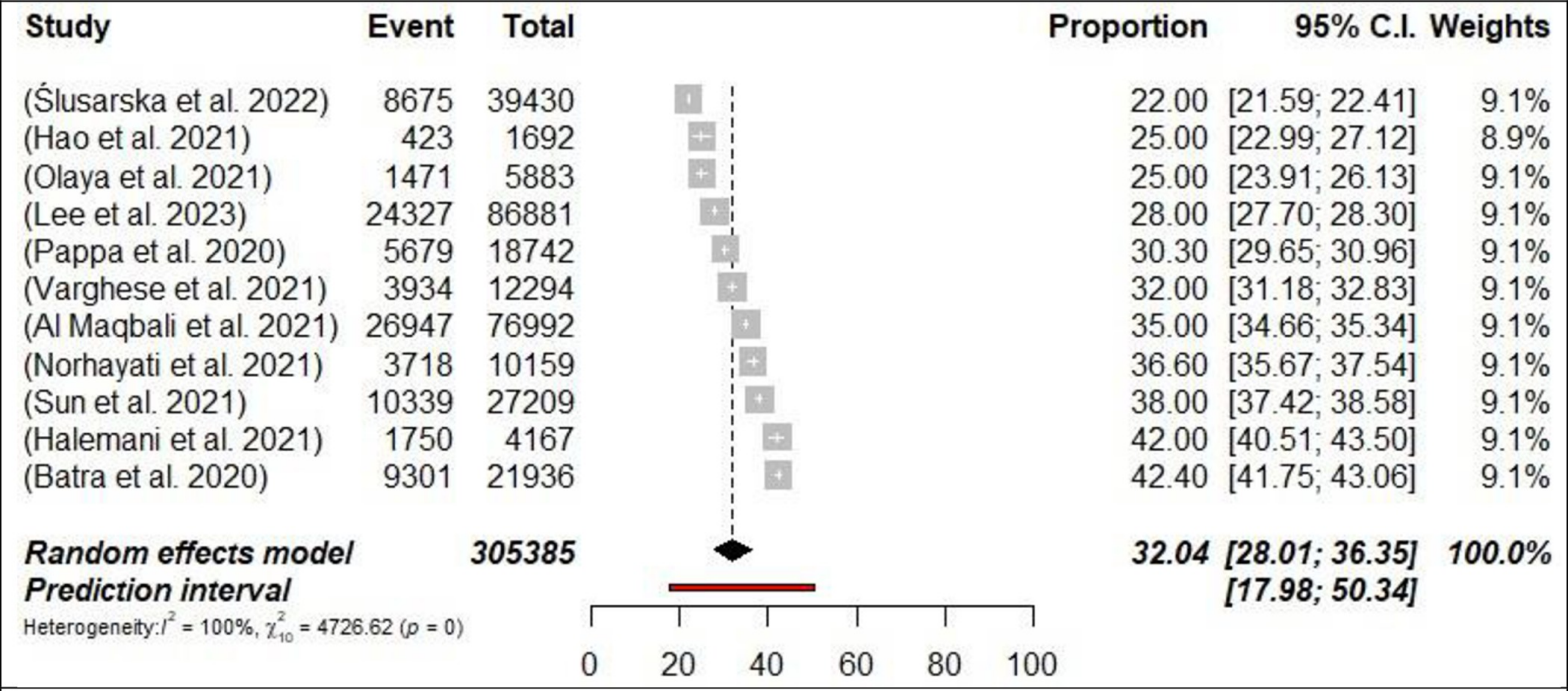
Forest plot of the prevalence of depression among nurses (N = 11).

**Fig 9:**
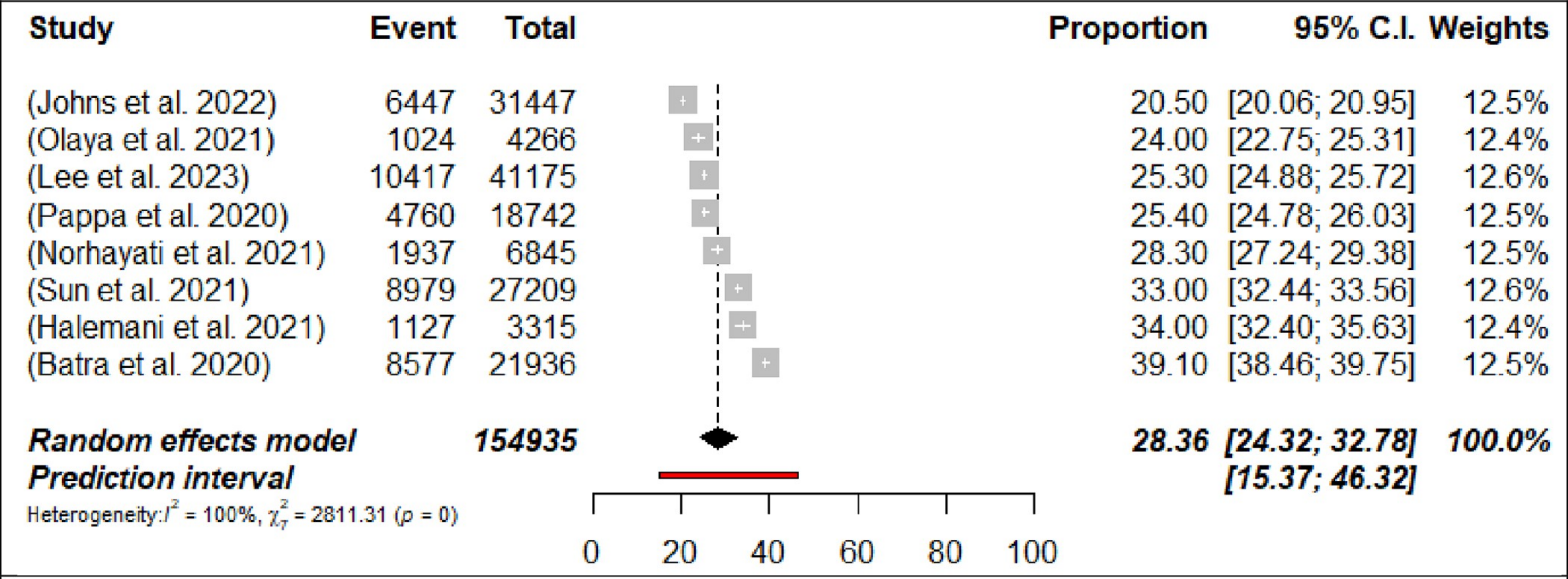
Forest plot of the prevalence of depression among physicians (N = 8).

A total of 54 meta-analyses examined the prevalence of depression among HCPs during the COVID-19 pandemic and results ranged from 14% [52] to 65.6% [58]. The pooled prevalence was 29.4% (698,808/2,349,613 participants, 95% CI 27.13–31.84) with 95% PI: 15.01–49.62 (Fig 7: Forest Plots) and there was a significant result in terms of the study heterogeneity (*p*< 0.0001, I^2^ = 99.9%). In subgroups analyses, the prevalence of depression was higher among nurses 32% (n = 11; 95% CI = 28–36.35, I^2^ = 99%) compared with physicians 28.4% (n = 8; 95% CI = 24.32–32.78, I^2^ = 99%) (Figs 8 and 9: Forest Plots). In the sensitivity analysis, the pooled prevalence remained stable when one meta-analysis was excluded at a time, with variations of less than 1%. Class III evidence revealed suggestive findings regarding the estimated prevalence of depression among HCPs, nurses, and physicians.

### 3.6 Prevalence of sleep disturbance

**Fig 10:**
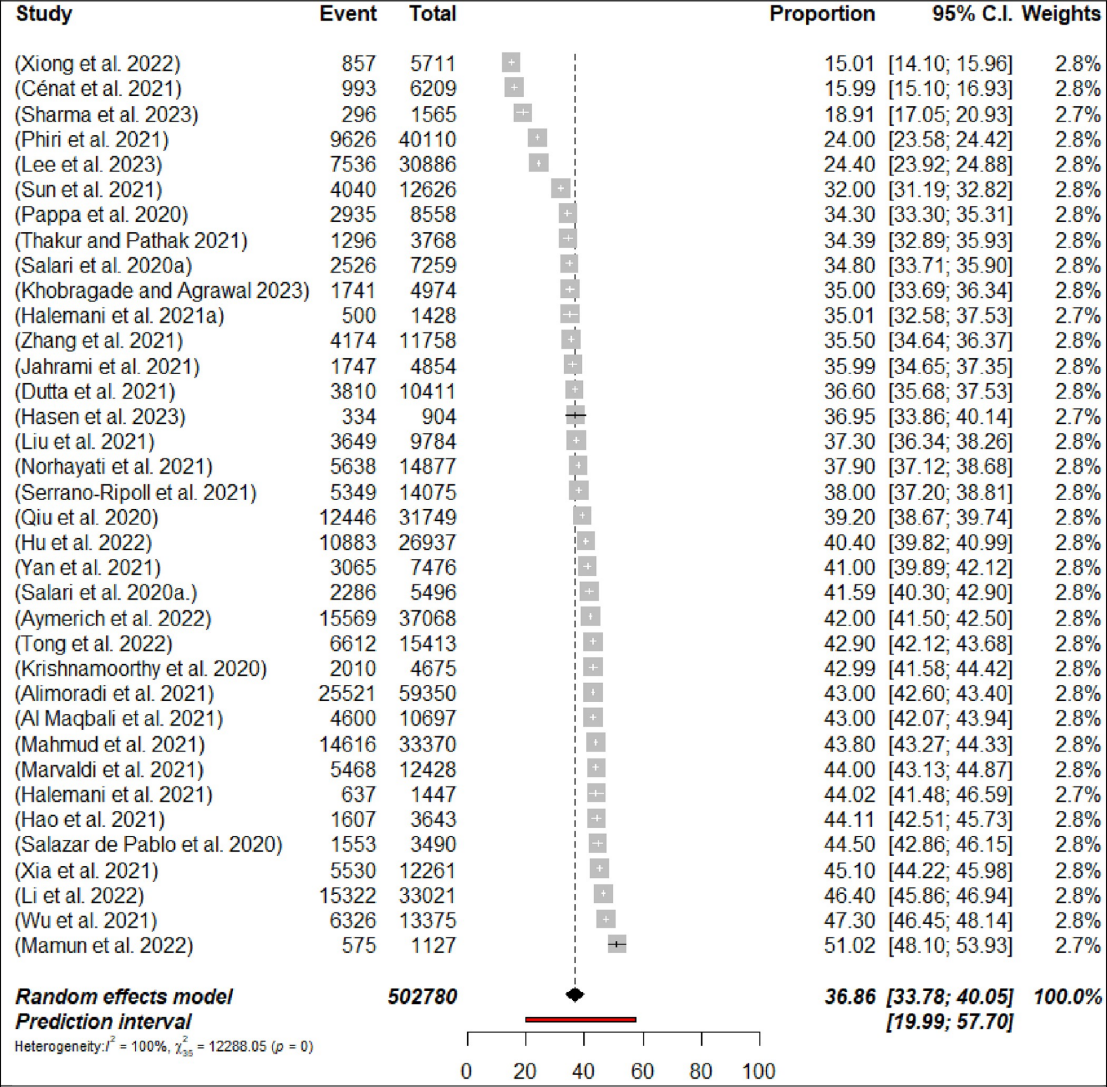
Forest plot of the prevalence of sleep disturbance among HCPs (N = 36).

**Fig 11:**
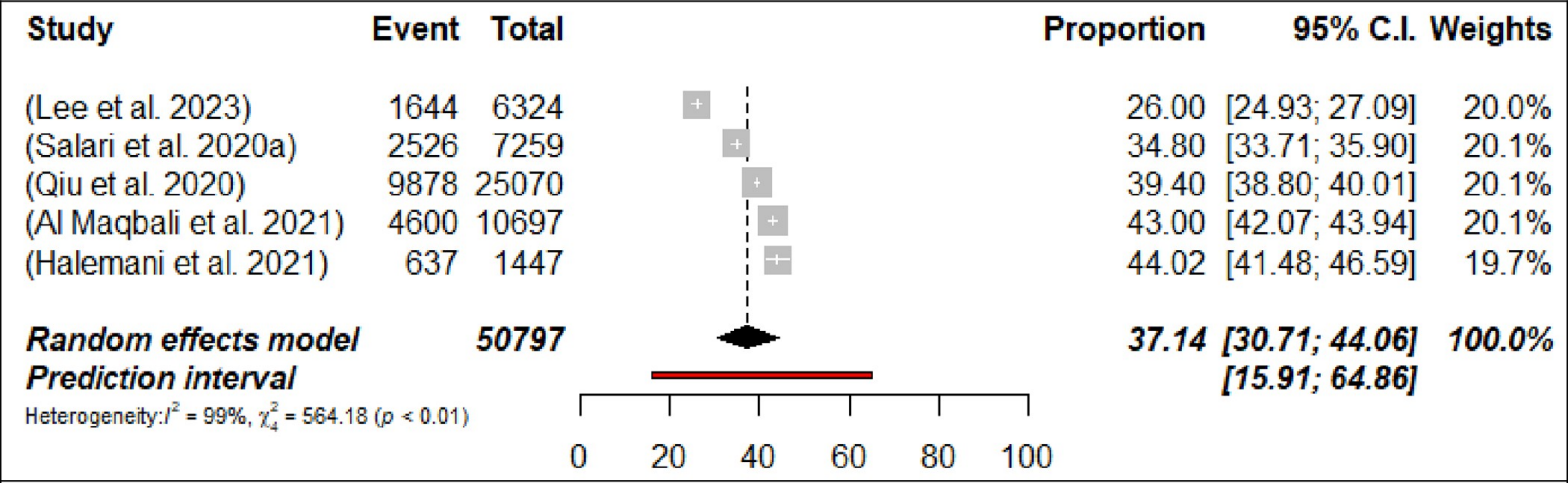
Forest plot of the prevalence of sleep disturbance among nurses (N = 5).

**Fig 12:**
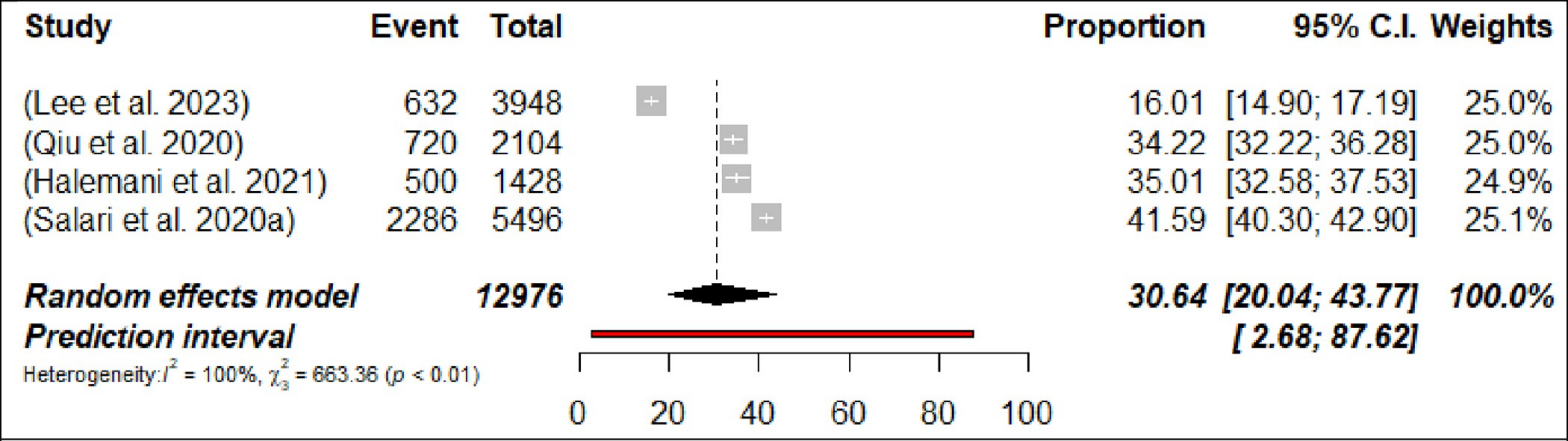
Forest plot of the prevalence of sleep disturbance among physicians (N = 4).

Sleep disturbance was assessed in 36 meta-analyses, with a calculated pooled prevalence of 36.9% (191,673/502,780 participants, 95% CI 33.78–40.05) with 95% PI: 19.99–57.70 (Fig 10: Forest Plots) with significant differences in terms of the meta-analyses heterogeneity presented (p< 0.0001, I^2^ = 99.7%). The prevalence of sleep disturbance ranged from 15.01% [93] to 47.3% [95]. In subgroup analyses, the prevalence of sleep disturbance was found to be higher among nurses at 37.1% (n = 5; 95% CI = 30.71–44.1, I^2^ = 99%) compared to physicians, where it was 30.6% (n = 4; 95% CI = 20.04–43.77, I^2^ = 99%) (Figs 11 and 12: Forest Plots). The estimated prevalence rate of sleep disturbance was deemed to be suggestive evidence (Class III). The pooled prevalence did not change in sensitivity analysis by excluding one meta-analyses each time by less than 3%.

### 3.7 Publication bias

The result of Egger’s regression test for all pooled prevalence indicates that publication bias was insignificant, showing no evidence of publication bias Table 2.

## 4. Discussion

To the best of our knowledge, this is the first umbrella review to provide a comprehensive synthesis of the estimate of the aggregate data prevalence symptoms of stress, anxiety, depression, and sleep disturbance among HCPs, physicians, and nurses during the entire COVID-19 pandemic.

In the present umbrella review, which utilizes aggregate data from 71 meta-analyses, the most prevalent problems among healthcare professionals (HCPs) were found to be stress (37%), followed by sleep disturbance (36.9%), anxiety (31.8%), and depression (29.4%). The findings among HCPs are slightly higher than the prevalence estimates from the general population (prevalence estimates of 27% for sleep disturbance; 36% for stress; 26% for anxiety and 28% for depression) during the COVID-19 pandemic [109]. In addition, a report by the World Health Organization prior to the COVID-19 pandemic estimated that the global prevalence of anxiety and depression was 4.4% and 3.6% respectively [110]. While some of this disparity may result from different methodological approaches used, the prevalence of depression and anxiety during the COVID-19 pandemic appears to have been higher than before the outbreak. The rise in mental health problems among HCPs may have been triggered by the uncertainty surrounding the pandemic, increased workload, and the fear of family transmission, any, or all of which may also contribute to the higher prevalence of these conditions.

The results of this umbrella review revealed higher prevalence rates compared with two previous reviews of meta-analyses [18,20]. These include 10 meta-analyses which reported prevalence rates during COVID-19 among HCPs: 25% for anxiety and 24% for depression [18]. Another umbrella review involving 18 meta-analyses found stress in 36% of the sample, depression in 26%, anxiety in 27% and sleep disturbance in 32% among HCPs during the COVID-19 pandemic [20]. It is important to highlight that the previous reviews included meta-analyses published before March 2021, while this current review included studies published until January 2024. As a result, the current umbrella review includes more meta-analyses compared to the two previous umbrella reviews [18,20]. This umbrella review therefore extends the scientific knowledge of the impact of COVID on mental health of HCPs.

The results of our study suggest that the psychological trauma experienced by HCPs during the SARS and MERS epidemics was lower than that experienced during the COVID-19 pandemic [111–114]. However, the difference between COVID-19 and previous pandemics could be explained by the high mortality rate and infectious potential of the COVID-19 pandemic. The results of this study suggest that the COVID-19 pandemic has had a significant negative effect on the psychological health of HCPs. One important lesson that should be learned is that early detection and treatment are carried out to prevent these types of psychological issues developing into more complex ones.

The result of this analysis demonstrates a higher level of anxiety, depression and sleep disturbance among nurses compared to physicians. One explanation for this may be that nurses are involved in more prolonged and closer contact to COVID-19 patients than physicians [115–117]. Another possible reason might be due to the higher number of nurses included in original studies.

The current review found that the overall pooled prevalence varied between the meta-analyses, for example ranging between 1% [72] and 66.6% [58] for stress, 11.% [40] and 71.9% [58] for anxiety, 14% [52] and 65.6% [58] for depression, and 15% [93] and 47.3% [95] for sleep disturbance. This could be linked to the varying COVID-19 infection and mortality rates in the countries in which the studies were conducted. Other possible reasons might relate to the healthcare system, cultural norms of HCPs, and their perceptions of stress, anxiety, depression, and sleep disturbance which in turn might be influenced by their working conditions, exposure to pandemics, intensity of lockdown and social distancing strategies, and perceived support. For instance, the result of the meta-analysis by El-Qushayri [58] showed the highest prevalent rate in terms of stress, anxiety and depression. This may be because this meta-analysis included only HCPs from Egypt, which in turn might indicate that the Egyptian healthcare system was severely affected by COVID-19 compared to other countries [118].

The finding of this umbrella review highlights significant negative effect that the COVID-19 pandemic has had on the psychological health of HCPs, further emphasizing the need for regular mental health assessment and management in this population. Due to the increasing number of complex traumas that HCPs are experiencing, special attention should be paid to the development of positive traumatic growth. The higher prevalence of stress, anxiety, depression, and sleep disturbance among HCPs have important implications for both the policies and practices of the healthcare system under consideration. It is important to identify effective interventions for HCPs such as the behavioural and educational interventions that have been suggested, including the development of a sense of coherence, positive thinking, and social support [119–121]. Currently there is a lack of evidence about the effectiveness of some psychological interventions that were adapted for use during COVID-19 pandemic specifically for healthcare workers [122,123].

Heterogeneity was significant in the majority of the analyses; several reasons can be attributed to this prominence. Firstly, the individual studies within each meta-analysis might differ substantially in terms of their design, sample sizes, interventions or exposures, and outcome measures. These variations can lead to differing effect sizes or conclusions, making the integration of results into a cohesive summary more complex. Furthermore, heterogeneity can arise from variations in the quality of these studies. While some research might have been meticulously conducted with strict inclusion criteria and rigorous methodologies, other studies on healthcare workers may have inherent biases or confounding factors due to the rapidly changing nature of the pandemic, the pressures of lockdowns, and their effects.

The unique characteristics and experiences of healthcare workers during the COVID-19 crisis, compounded by the challenges of lockdown measures, have the potential to further amplify this variability. Factors such as age, gender, ethnicity, and underlying health conditions, when combined with the stress, increased workload, and challenges of the pandemic and lockdown situations, can significantly influence study outcomes. Additionally, methodological differences in individual studies, like the use of a wide variety of questionnaires to measure symptoms, varied cut-off points, and severity thresholds, as well as the absence of a consistent ’gold standard’ for diagnostic interviews, can contribute to increased heterogeneity. In the context of the umbrella review, synthesizing findings from such a diverse collection of meta-analyses, particularly those focused on healthcare workers during this unparalleled period marked by fluctuating lockdown measures, poses a formidable challenge. Such complexity may constrain the robustness and precision of the conclusions drawn.

One of the most critical factors that policymakers need to consider when it comes to implementing effective interventions is the availability of organizational support. This can be done through various work-based interventions such as implementing shorter working hours and having buddy systems [124]. In addition, other measures such as providing mental health consultants and tele counselling can also help reduce the impact of the outbreak of disease on the well-being of staff members [125,126].

### 4.1 Limitations

Several limitations must be taken into consideration when interpreting the results of this umbrella review even though one strength of this methodology is that it provides comprehensive evidence regarding the mental health problems that were faced by HCPs during the COVID-19 pandemic, First, there is a possibility of selection bias. For example, non-English language meta-analyses were not included in this umbrella review, and this may introduce a selection bias. Second, it may be the case that some meta-analyses may have included the same primary studies and that there is consequently a significant study overlap between the meta-analyses included in this review. However, since the results of the studies were then combined with other studies, and a new result was presented, these were regarded as being new studies [25]. Further, several researchers address overlapping by removing some of the reviews with higher rates of overlapping [26,127]. Although removing the overlapping meta-analyses solves the problem of dependent effects, it might introduce a bias of its own. Excluding one of two overlapping meta-analyses from an umbrella review will bias the overall estimate [128,129]. In addition, Hennessy and Johnson [127] clearly mention that the overlap of primary studies included in a meta-review is not necessarily a bias but often can be a benefit.

Third, the various methodologies of the primary studies that were included in the meta-analyses, in terms of sampling methods, assessment tools, operational definitions of the symptoms and study length, might have affected the sensitivity and specificity with regard to detecting the prevalence estimations of stress, anxiety, depression, and sleep disturbance [130]. Finally, it should be noted that stress, anxiety, depression, and sleep disturbance varied between the HCPs studied. Therefore, future research should focus on the difference contexts of estimation prevalence between HCPs and should report the prevalence in each group.

## 5. Conclusion

In summary, this umbrella review systematically analyses the currently available evidence on the prevalence of stress, anxiety, depression, and sleep disturbance among HCPs in relation to COVID-19. It revealed that the incidence of these symptoms is high in the HCP population. However, there is wide variation in the degree of these conditions among this HCP population. This may be due to the varying experiences of COVID-19 and the cultural differences in the countries where the studies have been carried out. It is clear from the current evidence that strategies involving multi-level interventions are required to develop effective interventions that can help improve the mental health and well-being of HCPs and foster post-traumatic growth. Further research needs to address the limitations of the existing literature, in order to enable the authorities, providers, and patients to improve the quality of mental health on the part of HCPs.

## Supporting information

S1 Checklist PRISMA 2020 checklist. (DOCX)

S1 Table Quality assessment result of meta analysis using the AMSTAR-2 (N = 72). (DOCX)
