# Supplementary material for: Stress, anxiety, depression and sleep disturbance among healthcare professional during the COVID-19 pandemic: An umbrella review of 72 meta-analyses
Source: PLoS One. 2024 May 9;19(5):e0302597. doi: 10.1371/journal.pone.0302597 (PMC11081353; doi:10.1371/journal.pone.0302597)
Supplement: S1 Table — (DOCX) [file pone.0302597.s002.docx]

**Supplementary Table 1: Quality Assessment Result of Meta Analysis using the AMSTAR-2 (N=72):**

|  | **Study** | **1**  **(0-1)** | **2^*^**  **(0-2)** | **3**  **(0-1)** | **4^*^**  **(0-2)** | **5**  **(0-1)** | **6**  **(0-1)** | **7^*^**  **(0-2)** | **8**  **(0-2)** | **9^*^**  **(0-2)** | **10**  **(0-1)** | **11^*^**  **(0-1)** | **12**  **(0-1)** | **13^*^**  **(0-1)** | **14**  **(0-1)** | **15^*^**  **(0-1)** | **16**  **(0-1)** | **AMSTAR-2**  **Quality** |
| --- | --- | --- | --- | --- | --- | --- | --- | --- | --- | --- | --- | --- | --- | --- | --- | --- | --- | --- |
| 1 | (Batra et al. 2020) | Yes | Yes | No | P/ Yes | Yes | No | P/ Yes | Yes | P/ Yes | No | Yes | Yes | No | No | No | Yes | Moderate |
| 2 | (Krishnamoorthy et al. 2020) | Yes | P/ Yes | No | P/ Yes | Yes | No | Yes | P/ Yes | No | No | No | Yes | No | No | Yes | Yes | Moderate |
| 3 | (Pappa et al. 2020) | Yes | P/ Yes | No | Yes | No | P/ Yes | Yes | No | Yes | No | Yes | No | No | No | No | Yes | Moderate |
| 4 | (Qiu et al. 2020) | Yes | Yes | Yes | P/ Yes | Yes | Yes | Yes | P/ Yes | Yes | No | Yes | No | Yes | Yes | Yes | Yes | Low |
| 5 | (Salari et al. 2020a) | Yes | P/ Yes | No | P/ Yes | Yes | P/ Yes | Yes | P/ Yes | No | No | Yes | No | No | No | Yes | No | Critically Low |
| 6 | (Salari et al. 2020b) | Yes | P/ Yes | Yes | Yes | Yes | No | Yes | Yes | No | No | Yes | No | No | Yes | No | Yes | Critically Low |
| 7 | (Allan et al. 2020) | Yes | Yes | Yes | P/ Yes | Yes | P/ Yes | Yes | P/ Yes | P/ Yes | No | Yes | No | Yes | Yes | Yes | No | Critically Low |
| 8 | (Salazar de Pablo et al. 2020) | Yes | P/ Yes | No | P/ Yes | Yes | No | P/ Yes | P/ Yes | P/ Yes | No | Yes | No | P/ Yes | Yes | No | Yes | Moderate |
| 9 | (Ren et al. 2020) | Yes | Yes | Yes | P/ Yes | Yes | P/ Yes | Yes | P/ Yes | Yes | No | Yes | Yes | Yes | Yes | Yes | No | Low |
| 10 | (Pan et al. 2020) | Yes | Yes | Yes | P/ Yes | Yes | P/ Yes | Yes | Yes | Yes | No | Yes | No | Yes | Yes | Yes | Yes | Low |
| 11 | (Zhang et al. 2021) | Yes | Yes | Yes | P/ Yes | No | P/ Yes | No | Yes | Yes | No | Yes | Yes | Yes | Yes | Yes | Yes | Moderate |
| 12 | (Adibi et al. 2021) | Yes | Yes | Yes | P/ Yes | Yes | Yes | Yes | P/ Yes | Yes | No | Yes | No | Yes | Yes | Yes | Yes | Low |
| 13 | (Al Maqbali et al. 2021) | Yes | Yes | Yes | Yes | Yes | Yes | Yes | Yes | Yes | No | Yes | Yes | Yes | Yes | Yes | No | Moderate |
| 14 | (Alimoradi et al. 2021) | Yes | P/ Yes | Yes | Yes | Yes | Yes | Yes | Yes | P/ Yes | No | Yes | No | No | Yes | Yes | Yes | Low |
| 15 | (Bareeqa et al. 2021) | Yes | Yes | Yes | Yes | Yes | Yes | Yes | Yes | Yes | No | Yes | Yes | Yes | Yes | Yes | No | Low |
| 16 | (Cénat et al. 2021) | Yes | P/ Yes | No | Yes | No | P/ Yes | Yes | No | Yes | No | Yes | No | No | No | No | Yes | Moderate |
| 17 | (Ching et al. 2021) | Yes | Yes | Yes | P/ Yes | Yes | P/ Yes | Yes | P/ Yes | P/ Yes | No | Yes | No | Yes | Yes | Yes | Yes | Low |
| 18 | (Dutta et al. 2021) | Yes | Yes | P/ Yes | Yes | Yes | Yes | Yes | P/ Yes | Yes | No | Yes | No | No | No | Yes | Yes | Low |
| 19 | (Hao et al. 2021) | Yes | Yes | Yes | P/ Yes | Yes | P/ Yes | Yes | P/ Yes | Yes | No | Yes | No | Yes | Yes | Yes | Yes | Low |
| 20 | (Yan et al. 2021) | Yes | Yes | Yes | Yes | Yes | Yes | Yes | Yes | Yes | No | Yes | Yes | Yes | Yes | Yes | No | Low |
| 21 | (Liu et al. 2021) | Yes | Yes | Yes | Yes | Yes | Yes | Yes | Yes | Yes | No | Yes | Yes | Yes | Yes | Yes | No | Low |
| 22 | (Mahmud et al. 2021) | Yes | Yes | Yes | P/ Yes | Yes | P/ Yes | Yes | Yes | P/ Yes | No | Yes | No | Yes | Yes | Yes | Yes | Low |
| 23 | (Marvaldi et al. 2021) | Yes | P/ Yes | No | Yes | No | P/ Yes | Yes | No | Yes | No | Yes | No | No | No | No | Yes | Moderate |
| 24 | (Phiri et al. 2021) | Yes | Yes | Yes | Yes | Yes | Yes | Yes | Yes | Yes | No | Yes | Yes | Yes | Yes | Yes | No | Moderate |
| 25 | (Santabárbara et al. 2021) | Yes | Yes | Yes | P/ Yes | Yes | P/ Yes | Yes | Yes | P/ Yes | No | Yes | No | No | Yes | Yes | Yes | Low |
| 26 | (Saragih et al. 2021) | Yes | Yes | Yes | P/ Yes | Yes | P/ Yes | Yes | P/ Yes | P/ Yes | No | Yes | No | Yes | Yes | Yes | Yes | Low |
| 27 | (Singh et al. 2021) | Yes | Yes | Yes | P/ Yes | Yes | P/ Yes | Yes | Yes | Yes | No | Yes | No | Yes | No | Yes | No | Critically Low |
| 28 | (Sun et al. 2021) | Yes | Yes | P/ Yes | Yes | Yes | Yes | Yes | P/ Yes | Yes | No | Yes | No | No | No | Yes | Yes | Low |
| 29 | (Varghese et al. 2021) | Yes | Yes | Yes | P/ Yes | Yes | P/ Yes | Yes | P/ Yes | Yes | No | Yes | No | Yes | Yes | Yes | Yes | Low |
| 30 | (Wu et al. 2021) | Yes | No | No | P/ Yes | No | Yes | No | P/ Yes | P/ Yes | No | Yes | Yes | Yes | Yes | Yes | Yes | Moderate |
| 31 | (Xia et al. 2021) | Yes | P/ Yes | Yes | P/ Yes | Yes | Yes | Yes | Yes | Yes | No | Yes | No | Yes | Yes | Yes | Yes | Moderate |
| 32 | (Deng et al. 2021) | Yes | Yes | Yes | P/ Yes | Yes | P/ Yes | Yes | Yes | Yes | No | Yes | Yes | Yes | Yes | Yes | No | Low |
| 33 | (Dong et al. 2021) | Yes | P/ Yes | No | P/ Yes | Yes | No | Yes | P/ Yes | No | No | No | Yes | No | No | Yes | Yes | Moderate |
| 34 | (Serrano-Ripoll et al. 2021) | Yes | P/ Yes | No | Yes | No | P/ Yes | Yes | No | Yes | No | Yes | No | No | No | No | Yes | Moderate |
| 35 | (Hossain et al. 2021) | Yes | Yes | Yes | P/ Yes | Yes | P/ Yes | Yes | P/ Yes | Yes | No | Yes | No | Yes | Yes | Yes | Yes | Low |
| 36 | (Li et al. 2021) | Yes | Yes | Yes | P/ Yes | Yes | P/ Yes | Yes | Yes | Yes | No | Yes | Yes | Yes | Yes | Yes | No | Low |
| 37 | (El-Qushayri et al. 2021) | Yes | P/ Yes | No | P/ Yes | Yes | No | Yes | P/ Yes | No | No | No | Yes | No | No | Yes | Yes | Moderate |
| 38 | (Jahrami et al. 2021) | Yes | P/ Yes | No | Yes | No | P/ Yes | Yes | No | Yes | No | Yes | No | No | No | No | Yes | Moderate |
| 39 | (Olaya et al. 2021) | Yes | Yes | Yes | P/ Yes | Yes | P/ Yes | Yes | Yes | P/ Yes | No | Yes | No | Yes | Yes | Yes | Yes | Low |
| 40 | (Raoofi et al. 2021) | Yes | Yes | Yes | P/ Yes | Yes | Yes | Yes | Yes | P/ Yes | No | Yes | No | No | Yes | Yes | Yes | Low |
| 41 | (Salehi et al. 2021) | Yes | P/ Yes | No | Yes | No | P/ Yes | Yes | No | Yes | No | Yes | No | No | No | No | Yes | Moderate |
| 42 | (Abdulla et al. 2021) | Yes | Yes | Yes | Yes | Yes | Yes | Yes | Yes | Yes | No | Yes | Yes | Yes | Yes | Yes | No | Moderate |
| 43 | (Crocamo et al. 2021) | Yes | Yes | Yes | P/ Yes | Yes | P/ Yes | Yes | P/ Yes | P/ Yes | No | Yes | No | Yes | Yes | Yes | Yes | Low |
| 44 | (Halemani et al. 2021) | Yes | Yes | P/ Yes | Yes | Yes | Yes | Yes | P/ Yes | Yes | No | Yes | No | No | No | Yes | Yes | Low |
| 45 | (Hosen et al. 2021) | Yes | Yes | Yes | P/ Yes | Yes | P/ Yes | Yes | P/ Yes | Yes | No | Yes | No | Yes | Yes | Yes | Yes | Low |
| 46 | (Norhayati et al. 2021) | Yes | Yes | Yes | Yes | Yes | Yes | Yes | Yes | Yes | No | Yes | Yes | Yes | Yes | Yes | No | Low |
| 47 | (Zhao et al. 2021) | Yes | Yes | Yes | Yes | Yes | Yes | Yes | Yes | Yes | No | Yes | Yes | Yes | Yes | Yes | No | Low |
| 48 | (Thakur and Pathak 2021) | Yes | Yes | Yes | P/ Yes | Yes | P/ Yes | Yes | Yes | P/ Yes | No | Yes | No | Yes | Yes | Yes | Yes | Low |
| 49 | (Aymerich et al. 2022) | Yes | Yes | Yes | P/ Yes | Yes | Yes | Yes | Yes | P/ Yes | No | Yes | No | No | Yes | Yes | Yes | Low |
| 50 | (Hu et al. 2022) | Yes | Yes | Yes | P/ Yes | Yes | P/ Yes | Yes | P/ Yes | Yes | No | Yes | No | Yes | Yes | Yes | Yes | Low |
| 51 | (Johns et al. 2022) | Yes | Yes | Yes | P/ Yes | Yes | P/ Yes | Yes | Yes | Yes | No | Yes | Yes | Yes | Yes | Yes | No | Low |
| 52 | (Li et al. 2022) | Yes | Yes | Yes | P/ Yes | Yes | Yes | Yes | P/ Yes | Yes | No | Yes | No | Yes | Yes | Yes | Yes | Low |
| 53 | (Rezaei et al. 2022) | Yes | P/ Yes | Yes | Yes | Yes | Yes | Yes | Yes | P/ Yes | No | Yes | No | No | Yes | Yes | Yes | Low |
| 54 | (Xiong et al. 2022) | Yes | Yes | Yes | Yes | Yes | Yes | Yes | Yes | Yes | No | Yes | Yes | Yes | Yes | Yes | No | Low |
| 55 | (Zhang et al. 2022) | Yes | Yes | Yes | P/ Yes | Yes | P/ Yes | Yes | P/ Yes | Yes | No | Yes | Yes | Yes | Yes | Yes | No | Low |
| 56 | (Ślusarska et al. 2022) | Yes | Yes | Yes | P/ Yes | Yes | P/ Yes | Yes | Yes | Yes | No | Yes | No | Yes | Yes | Yes | Yes | Low |
| 57 | (Blasco-Belled et al. 2022) | Yes | Yes | No | Yes | Yes | Yes | No | Yes | Yes | No | Yes | No | Yes | Yes | Yes | Yes | Moderate |
| 58 | (Huang et al. 2022) | Yes | Yes | Yes | P/ Yes | Yes | Yes | Yes | P/ Yes | Yes | No | Yes | No | Yes | Yes | Yes | Yes | Low |
| 59 | (Tran et al. 2022) | Yes | P/ Yes | Yes | Yes | Yes | Yes | Yes | Yes | P/ Yes | No | Yes | No | No | Yes | Yes | Yes | Low |
| 60 | (Tong et al. 2022) | Yes | P/ Yes | No | Yes | Yes | Yes | Yes | Yes | P/ Yes | No | No | No | No | Yes | Yes | Yes | Moderate |
| 61 | (Andhavarapu et al. 2022) | Yes | Yes | No | Yes | Yes | Yes | No | Yes | Yes | No | Yes | No | Yes | Yes | Yes | Yes | Moderate |
| 62 | (Mamun et al. 2022) | Yes | Yes | No | Yes | Yes | Yes | No | Yes | P/ Yes | No | Yes | No | Yes | Yes | Yes | Yes | Moderate |
| 63 | (Cheung et al. 2022) | Yes | Yes | No | Yes | Yes | Yes | No | P/ Yes | P/ Yes | No | Yes | No | Yes | Yes | Yes | Yes | Moderate |
| 64 | (Hasen et al. 2023b) | Yes | P/ Yes | No | Yes | Yes | Yes | Yes | Yes | P/ Yes | No | No | No | No | Yes | Yes | Yes | Moderate |
| 65 | (Athe et al. 2023) | Yes | Yes | Yes | P/ Yes | Yes | Yes | Yes | Yes | Yes | No | Yes | No | Yes | Yes | Yes | No | Critically Low |
| 66 | (Sialakis et al. 2023) | Yes | Yes | No | Yes | Yes | Yes | No | Yes | Yes | No | Yes | No | Yes | Yes | Yes | Yes | Moderate |
| 67 | (Gheshlagh et al. 2023) | Yes | P/ Yes | No | Yes | Yes | Yes | Yes | Yes | P/ Yes | No | No | No | No | Yes | Yes | Yes | Moderate |
| 68 | (Wang et al. 2023) | Yes | Yes | No | Yes | Yes | Yes | No | Yes | Yes | No | Yes | No | Yes | Yes | Yes | Yes | Moderate |
| 69 | (Khobragade and Agrawal 2023) | Yes | Yes | Yes | P/ Yes | Yes | P/ Yes | Yes | P/ Yes | P/ Yes | No | Yes | No | Yes | Yes | Yes | No | Critically Low |
| 70 | (Lee et al. 2023) | Yes | Yes | No | Yes | Yes | Yes | No | Yes | P/ Yes | No | Yes | No | Yes | Yes | Yes | Yes | Moderate |
| 71 | (Sharma et al. 2023) | Yes | P/ Yes | No | Yes | Yes | Yes | Yes | Yes | P/ Yes | No | No | No | No | Yes | Yes | Yes | Moderate |
| 72 | (Hasen et al. 2023a) | Yes | Yes | Yes | Yes | Yes | Yes | No | Yes | Yes | No | Yes | No | Yes | Yes | Yes | Yes | Moderate |

* Critical Domains

1. Did the research questions and inclusion criteria for the review include the components of PICO? (Yes/No).
2. Did the report of the review contain an explicit statement that the review methods were established prior to the conduct of the review and did the report justify any significant deviations from the protocol? (Yes/Partial Yes/No).
3. Did the review authors explain their selection of the study designs for inclusion in the review? (Yes/No).
4. Did the review authors use a comprehensive literature search strategy? (Yes/Partial Yes/No).
5. Did the review authors perform study selection in duplicate? (Yes/No).
6. Did the review authors perform data extraction in duplicate? (Yes/No).
7. Did the review authors provide a list of excluded studies and justify the exclusions? (Yes/Partial Yes/No).
8. Did the review authors describe the included studies in adequate detail? (Yes/Partial Yes/No).
9. Did the review authors use a satisfactory technique for assessing the risk of bias (RoB) in individual studies that were included in the review? (Yes/Partial Yes/No).
10. Did the review authors report on the sources of funding for the studies included in the review? (Yes/No).
11. If meta-analysis was performed did the review authors use appropriate methods for statistical combination of results? (Yes/No).
12. If meta-analysis was performed, did the review authors assess the potential impact of RoB in individual studies on the results of the meta-analysis or other evidence synthesis? (Yes/No).
13. Did the review authors account for RoB in individual studies when interpreting/ discussing the results of the review? (Yes/No).
14. Did the review authors provide a satisfactory explanation for, and discussion of, any heterogeneity observed in the results of the review? (Yes/No).
15. If they performed quantitative synthesis did the review authors carry out an adequate investigation of publication bias (small study bias) and discuss its likely impact on the results of the review? (Yes/No).
16. Did the review authors report any potential sources of conflict of interest, including any funding they received for conducting the review? (Yes/No).

Shea BJ, Reeves BC, Wells G, Thuku M, Hamel C, Moran J, et al. AMSTAR 2: a critical appraisal tool for systematic reviews that include randomised or non-randomised studies of healthcare interventions, or both. BMJ. 2017;358: j4008. doi:10.1136/bmj.j4008
