# Supplementary material for: Stress, anxiety, depression and sleep disturbance among healthcare professional during the COVID-19 pandemic: An umbrella review of 72 meta-analyses
Source: PLoS One. 2024 May 9;19(5):e0302597. doi: 10.1371/journal.pone.0302597 (PMC11081353; doi:10.1371/journal.pone.0302597)
Supplement: Attachment — Submitted filename: PONE-D-24-02790_revision requests.docx [file pone.0302597.s003.docx]

Thank you for inviting me to review this interesting umbrella review focused on mental health impacts of COVID-19 pandemic on healthcare professionals. The review is well organized, and it provides comprehensive evidence on the prevalence of mental disorders among HCPs during the COVID-19 pandemic.

A. One of the basic matters in this review is the inclusion of 17 meta-analyses that included a mixed population (General and HCPs-Page 10 line 8) while the remaining studies focus on HCPs only. In this case it is very hard to reach a concrete conclusion for HCPs since significant number of included studies focus on non-healthcare population. Authors need to address this issue at first and most.
B. The number of studies included in the umbrella review mentioned in the title and displayed in the PRISMA diagram (i.e. 71) and stated in the abstract section (i.e. 59) needs correction.
C. A study conducted by Hassen AA and et als (Anxiety and stress among healthcare professionals during COVID-19 in Ethiopia: systematic review and meta-analysis. BMJ Open. 2023, 13 (2) e070367; <https://doi.org/10.1136/bmjopen-2022-070367>) is missed from your included studies list which could be another finest input for the current review.
D. The study becomes more comprehensive if the factors associated with the mentioned mental health problems were also provided following a meta regression analysis if possible.

Additional revisions needed for further improving the current manuscript are provided below separately for each section.

**Abstract**

a. Correct spelling for CHINAL,
b. Correct the number (*Fifty-nine meta-analyses involved 2,308 primary studies were included after a full-text review*.) of meta analyses involved as mentioned in the title and PRISMA diagram.
c. The authors stated that “In subgroup analyses the prevalence of anxiety and depression was higher among nurses than among physicians.” But from the result section the prevalence of sleep disturbance is also higher among nurses than among physicians (Page 12, line 6-8). Therefore, revise the above sentence to “**In subgroup analyses the prevalence of anxiety, depression and sleep disturbance was higher among nurses than among physicians**.”

**Introduction**

a. Well described and adequate information included.

**Methods**

Study selection

a. Authors have mentioned the inclusion and exclusion criteria. BUT what about studies that include both general population and HCPs? From your result section 17 studies included mixed population. (page 10 line 8)

**Result**

a. Well described
b. Any subgroup analysis for stress??

Slee disturbance

c. Page 12 line 111-12 -“The subgroup analysis was not performed due to an insufficient number of meta-analyses”. Contradicts with the above sentence (page 12 line 6-8) please resolve the paradox.

**Discussion and limitations**

a. Well, argued.

**Annexes**

A. Spelling and technical correction needed. (Figure 3. Forest Plot of the Prevalence of Stress Among HCPs Anxiety (N=54)
