# Supplementary material for: Stress, anxiety, depression and sleep disturbance among healthcare professional during the COVID-19 pandemic: An umbrella review of 72 meta-analyses
Source: PLoS One. 2024 May 9;19(5):e0302597. doi: 10.1371/journal.pone.0302597 (PMC11081353; doi:10.1371/journal.pone.0302597)
Supplement: Attachment — Submitted filename: Response to Reviewers.docx [file pone.0302597.s004.docx]

**Response to Reviewers:**

Dear Editor:

Thank you for your review, and I have addressed your comments accordingly.

Below are the revisions and changes made to the manuscript as per the reviewers' suggestions:

|  | Comments | Responses |
| --- | --- | --- |
|  | **Reviewer #1**  This is an interesting paper and the content is very good but I suggest the paper can be improved in the following ways: | We deeply appreciate your encouraging feedback on our umbrella review. Your positive comments on our work's organization and comprehensiveness are invaluable. Your input validates our efforts and guides us to enhance the manuscript further |
| 1 | -Please correct all parts of the article according to the guidelines of the journal authors guideline | The necessary modifications have been made to align with the journal guidelines. |
| 2 | Abstract  -In the conclusion part, it is necessary to specify the researcher's proposal to improve the conditions and use of the beneficiaries | An additional sentence to the conclusion was added.  “Such interventions may include the provision of mental health support services, such as counseling and peer support programs, as well as the implementation of organizational strategies to reduce workplace stressors.” |
| 3 | Introduction  Please bring the following items   1- Definition of the research problem 2- The magnitude and importance of the study variable 3- Expressing the necessity of conducting the study 4- Finally, the practical purpose of the study should be stated | **Definition of the research problem:**  The introduction outlines how healthcare professionals (HCPs) are vulnerable to stress, anxiety, depression, and sleep disturbances, especially during pandemics like COVID-19, drawing on previous outbreaks such as SARS and MERS. Furthermore, specific definitions for the mental health symptoms being studied (e.g., stress, anxiety, depression, sleep disturbance) are provided.  **Magnitude and importance of the study variable:**  The historical context of mental health issues among HCPs during outbreaks is highlighted, emphasizing their impact on individual well-being, healthcare system quality, and patient outcomes.  **Expressing the necessity of conducting the study:**  The introduction articulates the necessity of conducting the study by outlining the significant challenges faced by HCPs during the COVID-19 pandemic, such as increased workload, emotional strain, and risk of infection. Emphasis is placed on the importance of understanding the prevalence of mental health symptoms among HCPs to develop strategies for support and intervention, thereby safeguarding both the mental well-being of HCPs and the quality of healthcare services provided.  **Practical purpose of the study:**  The aim is to conduct an umbrella review to quantify meta-analytic findings regarding the prevalence of stress, anxiety, depression, and sleep disturbance among HCPs during the COVID-19 pandemic. This comprehensive analysis of existing literature aims to inform policy decisions, improve clinical risk predictions, and guide future research priorities.  Example of sentences with highlighted below:  In addition, several researchers have shown that work-related psychological disorders, including stress, anxiety, depression, and burnout, had already negatively affected the healthcare system before the COVID-19 pandemic, leading to low-quality care and high malpractice litigation [3–5].  It is important to understand the effects of the pandemic on the mental health and well-being of HCPs in order to help plan strategies to prevent these individuals from experiencing detrimental effects, and to ensure that they can continue to deliver healthcare services.  A high prevalence of mental health problems can adversely impact the quality of life of HCPs, increase disability, turnover, absenteeism, and errors, and can deleteriously affect patient outcomes which may lead to low patient satisfaction [13]. Further, it might increase suicidal ideation or self-harming among HCPs [14].  Since their publication, further meta-analyses have estimated the prevalence of stress, anxiety, depression, and sleep disturbance during the COVID-19 pandemic. The advantages of umbrella reviews include their ability to provide a comprehensive analysis of the literature, in this case about the prevalence of various mental disorders in HCPs during the COVID-19 pandemic. In addition, the results can then be used to make policy-level decisions to improve the quality of clinical care in terms of making clinical risk predictions and can inform future research priorities. |
| 4 | Methods  Please add the qualitative review for the selected final manuscript | A supplementary Table 1 presenting the Quality Assessment Results of the Meta-Analysis using the AMSTAR-2 (N=72) was added. |
| 5 | Discussion  In the discussion section, it is necessary to compare the main results of the study with the results of other studies in this field.  Please refer to published articles and used some reference to citation we proposed this article for enrichment.  -Anxiety, stress and depression levels among nurses of educational hospitals in Iran: Time of performing nursing care for suspected and confirmed COVID-19 patients  -Nursing Students' Competency to Attend Disaster Situations: A Study in Western Iran  - Post-traumatic stress disorder in medical workers involved in earthquake response: A systematic review and meta-analysis  - Prevalence of workplace violence against health care workers in hospital and pre-hospital settings: An umbrella review of meta-analyses  - Providing telenursing care for victims: a simulated study for introducing of possibility nursing interventions in disasters | Thank you for your feedback. We have carefully reviewed the comments and have incorporated references to published articles in the discussion section to compare the main results of our study with those of other studies in the field. |
|  |  |  |
|  | **Reviewer #2**  Reviewer #2: The authors present an umbrella review to quantify meta analytic findings aimed at estimating the prevalence of symptoms of stress, anxiety, depression, and sleep disturbance among HCPs during the COVID-19 pandemic. However, clarifications and modifications are needed in the manuscript. | We sincerely appreciate your thoughtful and encouraging feedback on our umbrella review. Your positive remarks regarding the organization and comprehensiveness of our work are highly valued. Your feedback not only validates our efforts but also provides invaluable guidance for further improving the manuscript. We are truly grateful for your contribution to enhancing the quality of our research. |
| 1 | The authors need to clarify whether 71 meta-analyses were included as they stated in the title and at the beginning of the results, or were "Fifty-nine meta-analyses involved 2,308 primary studies were included after a full-text review.", as they stated in the abstract? | Modified to be.  "Seventy-one meta-analyses involved 2,308 primary studies were included after a full-text review." |
| 2 | Furthermore, in table 1 the authors must add a column where they will list which instruments were used in the 71 systematic reviews included to diagnose the outcomes: stress, anxiety, depression and sleep disorders. | Given the nature of an umbrella review, where we synthesize findings from multiple meta-analyses, it becomes challenging to list the specific instruments used in each primary study within the included meta-analyses. However, we have provided a comprehensive overview of the methodologies employed across the meta-analyses in our manuscript Quality Appraisal section and supplementary Table 1. |
|  |  |  |
|  | **Reviewer #3**  Thank you for inviting me to review this interesting umbrella review focused on mental health impacts of COVID-19 pandemic on healthcare professionals. The review is well organized, and it provides comprehensive evidence on the prevalence of mental disorders among HCPs during the COVID-19 pandemic. | Thank you very much for your thoughtful and encouraging feedback on our umbrella review. We greatly appreciate your positive comments regarding the organization and comprehensiveness. Your feedback serves as valuable validation of our efforts and will certainly guide us in further improving the manuscript. We are grateful for your contribution to enhancing the quality of our work. |
| 1 | A. One of the basic matters in this review is the inclusion of 17 meta-analyses that included a mixed population (General and HCPs-Page 10 line 8) while the remaining studies focus on HCPs only. In this case it is very hard to reach a concrete conclusion for HCPs since significant number of included studies focus on non-healthcare population. Authors need to address this issue at first and most. | Thank you for bringing this to our attention. We acknowledge that our inclusion and exclusion criteria were specified in the manuscript. However, regarding meta-analyses that included both the general population and healthcare professionals (HCPs), such studies were indeed included in our umbrella review. We considered studies that encompassed healthcare professionals within their sample alongside the general population, as long as the data specific to healthcare professionals were extractable and relevant to our research objectives.  We modified the inclusion criteria section to reflect this:  ‘Presented results for HCPs as a group or separately (e.g., nurses or physicians only); further, studies involving non-HCPs must have presented results for HCPs separately and not pooled with non-HCPs.’  In the results section, we identified 17 studies that incorporated a mixed population. Among these studies, those that provided data specifically related to healthcare professionals were included in our umbrella review analysis. We ensured that only data pertinent to our study's focus on nurses and physicians were included in our synthesis and interpretation of findings. |
| 2 | B. The number of studies included in the umbrella review mentioned in the title and displayed in the PRISMA diagram (i.e. 71) and stated in the abstract section (i.e. 59) needs correction. | Modified to be.  "Seventy-one meta-analyses involved 2,308 primary studies were included after a full-text review." |
| 3 | C. A study conducted by Hassen AA and et als (Anxiety and stress among healthcare professionals during COVID-19 in Ethiopia: systematic review and meta-analysis. BMJ Open. 2023, 13 (2) e070367; https://doi.org/10.1136/bmjopen-2022-070367) is missed from your included studies list which could be another finest input for the current review. | We included this meta-analysis and made modifications throughout the manuscript. |
| 4 | D. The study becomes more comprehensive if the factors associated with the mentioned mental health problems were also provided following a meta regression analysis if possible. | It is important to acknowledge that the meta-regression will only involve factors such as the analysis software, the model used for analyses, and the quality appraisal tool (AMSTAR-2). It's worth noting that these factors may not be particularly beneficial or relevant to the reader's interests. However, if the reviewer suggests conducting regression analysis with these factors, we are open to considering it. |
|  |  |  |
| 5 | Abstract  a. Correct spelling for CHINAL, | Modified |
| 6 | b. Correct the number (Fifty-nine meta-analyses involved 2,308 primary studies were included after a full-text review.) of meta analyses involved as mentioned in the title and PRISMA diagram. | Modified to be.  "Seventy-one meta-analyses involved 2,308 primary studies were included after a full-text review." |
| 7 | c. The authors stated that “In subgroup analyses the prevalence of anxiety and depression was higher among nurses than among physicians.” But from the result section the prevalence of sleep disturbance is also higher among nurses than among physicians (Page 12, line 6-8). Therefore, revise the above sentence to “In subgroup analyses the prevalence of anxiety, depression and sleep disturbance was higher among nurses than among physicians.” | Sentences were modified.  In the discussion section:  ‘The result of this analysis demonstrates a higher level of anxiety, depression and sleep disturbance among nurses compared to physicians.’ |
| 8 | **Introduction**   a. Well described and adequate information included. | Thank you |
| 9 | Methods  Study selection  a. Authors have mentioned the inclusion and exclusion criteria. BUT what about studies that include both general population and HCPs? From your result section 17 studies included mixed population. (page 10 line 8) | For more clarity this sentence and an additional sentence were added  (Only data specifically related to healthcare professionals were included in the umbrella review analysis.) |
|  |  |  |
| 10 | Result   a. Well described | Thank you |
| 11 | b. Any subgroup analysis for stress?? | I have included nurses as subgroups, given the inadequate availability of meta-analyses for physicians.  Additional sentences were added in the result section.  In the subgroup analysis, the prevalence of anxiety among nurses was determined to be 42.6% (n=5; 95% CI=30.49-55.27, I^2^=99%), as shown in Figure 3: Forest Plots. However, the analysis for physicians was not conducted due to an insufficient number of available meta-analyses. |
| 12 | Sleep disturbance.   c. Page 12 line 111-12 -“The subgroup analysis was not performed due to an insufficient number of meta-analyses”. Contradicts with the above sentence (page 12 line 6-8) please resolve the paradox. | The sentences were omitted.  “The subgroup analysis was not performed due to an insufficient number of meta-analyses” |
| 13 | Discussion and limitations  a. Well, argued. | Thank you |
| 14 | Annexes  A. Spelling and technical correction needed. (Figure 3. Forest Plot of the Prevalence of Stress Among HCPs Anxiety (N=54) | Modified |
